# Supplementary material for: Interplay between Nonsense-Mediated mRNA Decay and DNA Damage Response Pathways Reveals that Stn1 and Ten1 Are the Key CST Telomere-Cap Components
Source: Cell Rep. 2014 May 15;7(4):1259–69. doi: 10.1016/j.celrep.2014.04.017 (PMC4518466; doi:10.1016/j.celrep.2014.04.017)
Supplement: Document S1. Supplemental Experimental Procedures and Figures S1–S5 [file mmc1.pdf]

Cell Reports, Volume *7*

Supplemental Information

Interplay between Nonsense-Mediated mRNA Decay and DNA Damage Response Pathways Reveals that Stn1 and Ten1 Are the Key CST Telomere-Cap Components

Eva-Maria Holstein, Kate R.M. Clark, and David Lydall

Figure S1

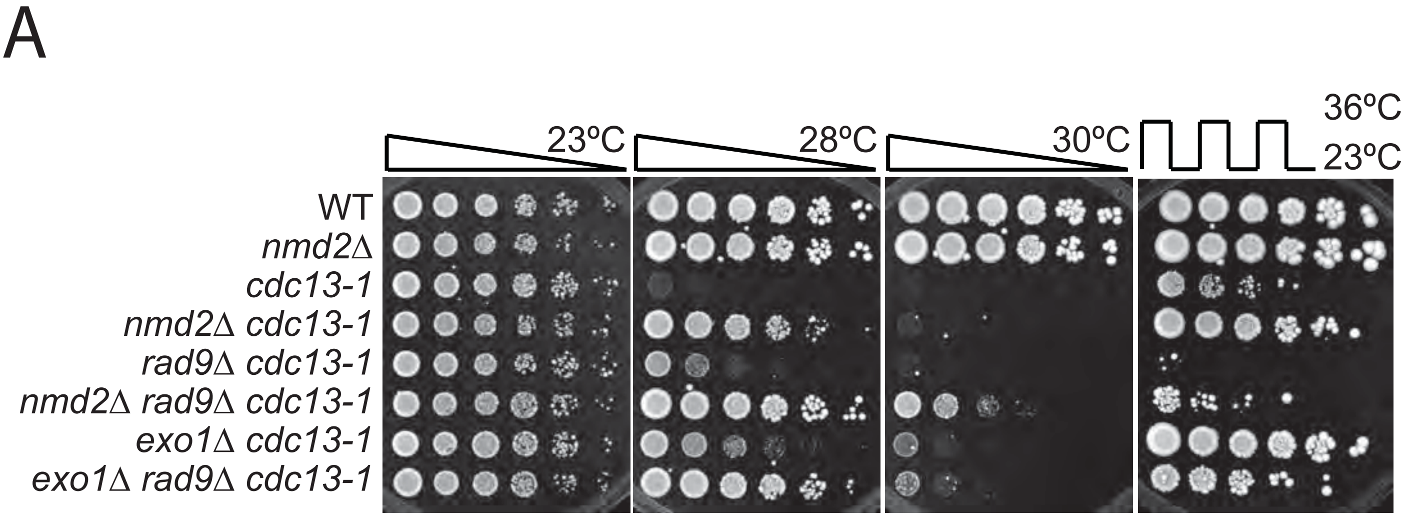
A
23ºC
28ºC
30ºC
36ºC
23ºC
WT
nmd2Δ
cdc13-1
nmd2Δ cdc13-1
rad9Δ cdc13-1
nmd2Δ rad9Δ cdc13-1
exo1Δ cdc13-1
exo1Δ rad9Δ cdc13-1

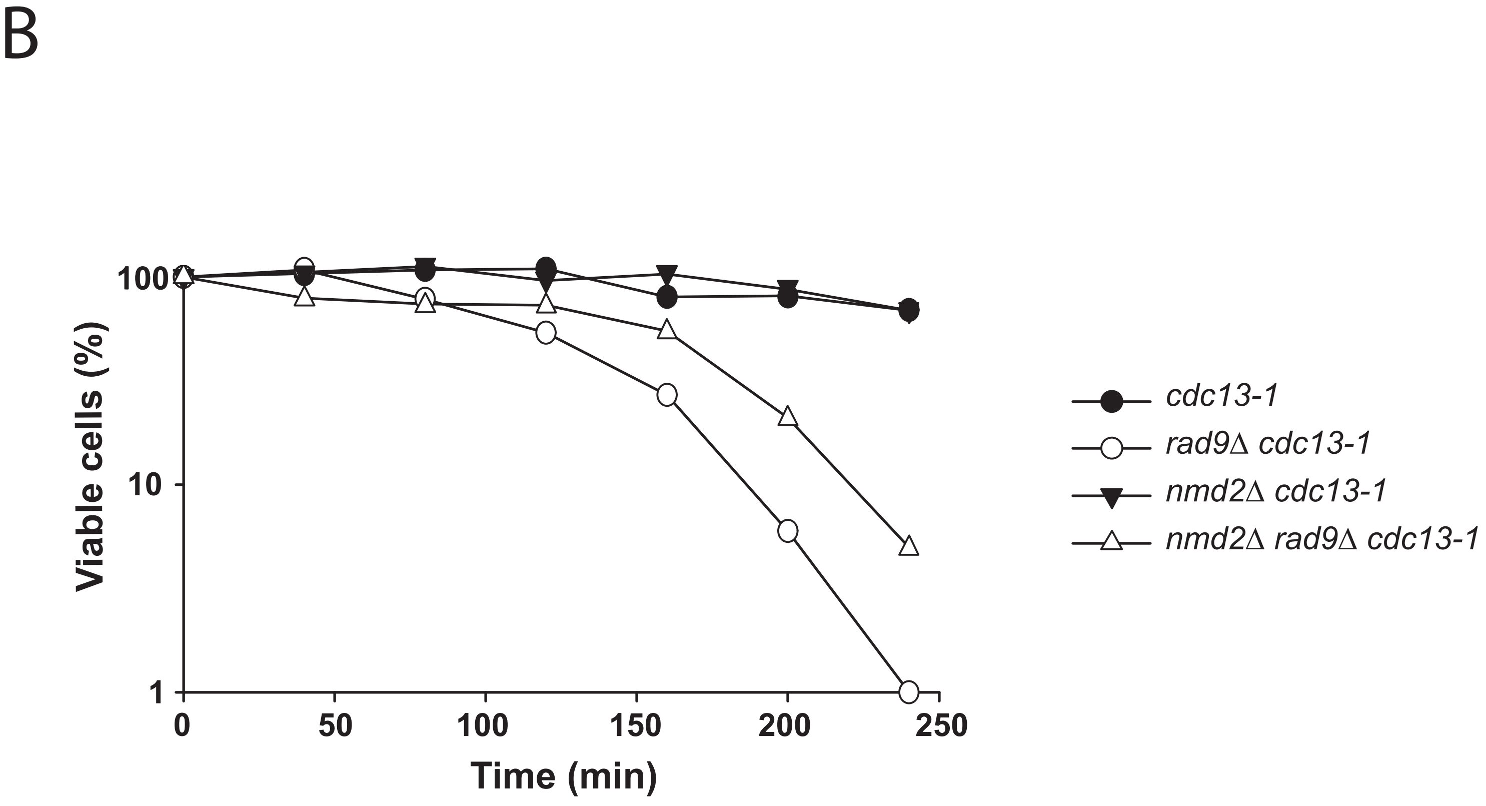
B
Viable cells (%)
100
10
1
0
50
100
150
200
250
Time (min)
cdc13-1
rad9Δ cdc13-1
nmd2Δ cdc13-1
nmd2Δ rad9Δ cdc13-1

Figure S1. Deletion of *NMD2* increases viability of *rad9*Δ *cdc13-1* mutants. Related to Figure 2.

(A) Saturated cultures were serially diluted across agar plates containing synthetic medium and were either grown at the temperature indicated for 3 days or cycled 3 times from 23°C for 4 hours to 36°C for 4 hours and then allowed to form colonies at 23°C for 3 days before being photographed. (B) Exponentially dividing cells containing *bar1*Δ and *cdc15-2* mutation were arrested at 23°C in G1 using alpha factor. Cultures were released from alpha factor and shifted to 36°C to induce telomere uncapping. Samples were taken at indicated time points, diluted, plated onto YEPD in duplicate and grown at 23°C for 3 days. Colony forming units were counted and viability was calculated using the dilution factor.

Figure S2

A *cdc13Δ/CDC13 nmd2Δ/NMD2 exo1Δ/EXO1 rad24Δ/RAD24*

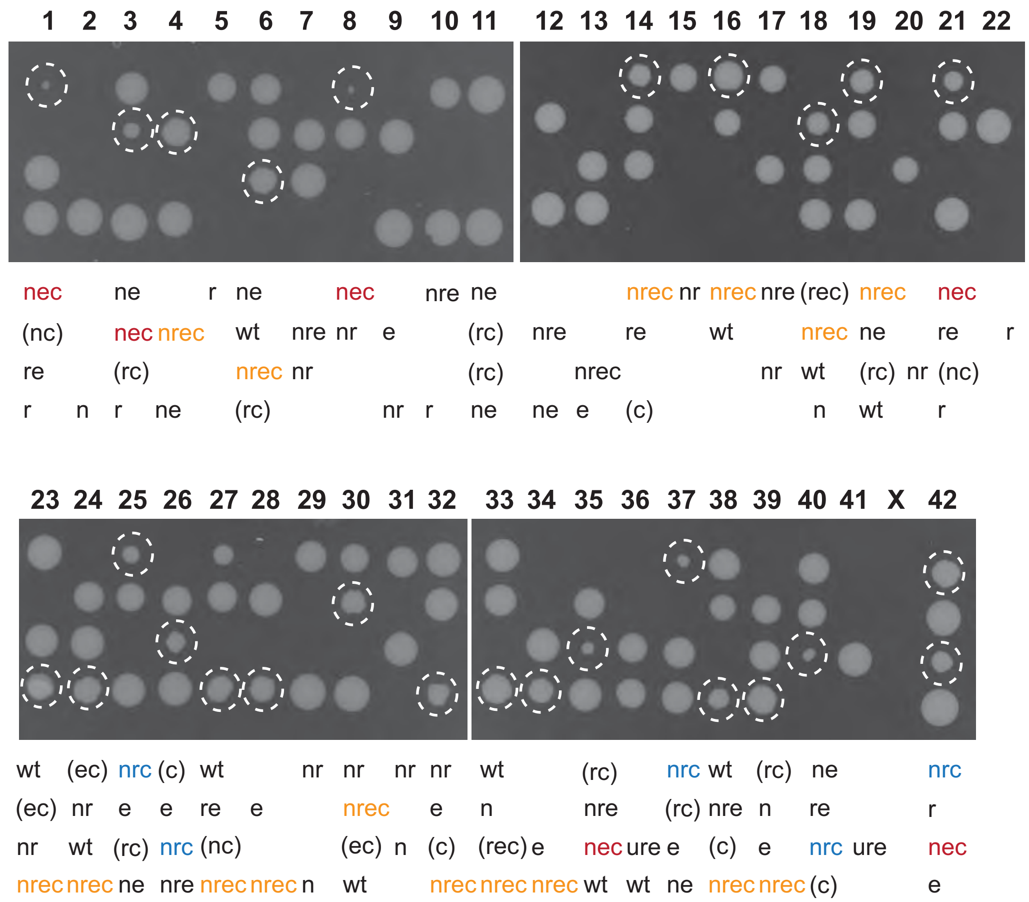

= viable *cdc13Δ* strain
n = *nmd2Δ*
r = *rad24Δ*
e = *exo1Δ*
c = *cdc13Δ*
() = inferred genotype
X = no spores dissected or spores not separated or spores lost

Genotype	Viability
cdc13Δ	0/4
nmd2Δ cdc13Δ	0/3
rad24Δ cdc13Δ	0/9
exo1Δ cdc13Δ	0/3
rad24Δ exo1Δ cdc13Δ	0/2
nmd2Δ rad24Δ cdc13Δ	5/5
nmd2Δ exo1Δ cdc13Δ	6/6
nmd2Δ rad24Δ exo1Δ cdc13	16/16

B

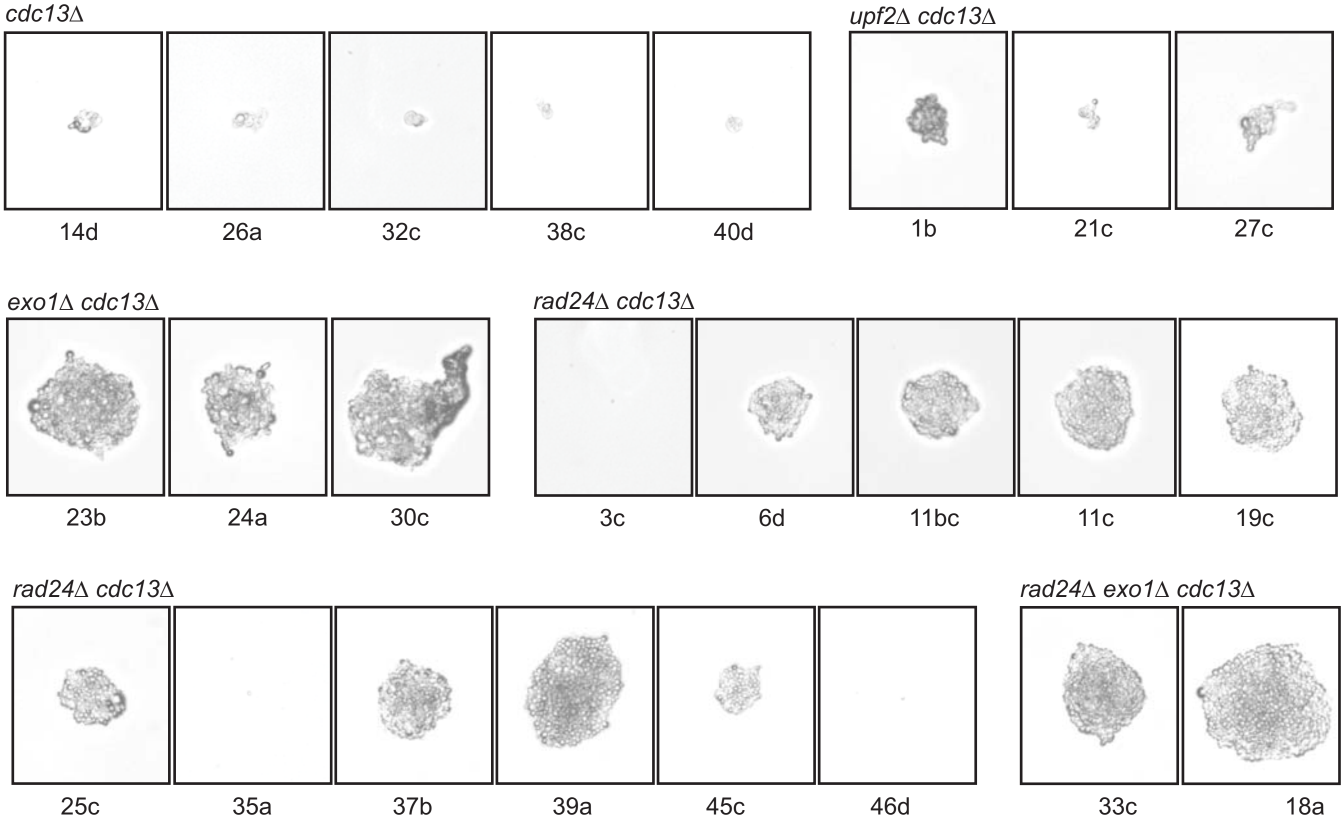

Figure S2. Growth of *cdc13*Δ strains. Related to Figure 3.

(A) Diploids containing heterozygous deletion mutations of *EXO1, NMD2, RAD24* and *CDC13* were sporulated. Tetrads were dissected and germinated on YEPD plates. Spores were allowed to form colonies for 5 days at 23°C before being photographed. Genotypes were determined by growth on appropriate selective plates. (B) Following germination of dissected diploids containing heterozygous deletion mutations of *EXO1, NMD2, RAD24* and *CDC13*, non-viable mutants were analyzed using a 20x objective on a Microtec microscope. An image was taken of each microcolony using a Jenoptik camera and all images displayed are reproduced at the same scale for direct comparison.

Figure S3

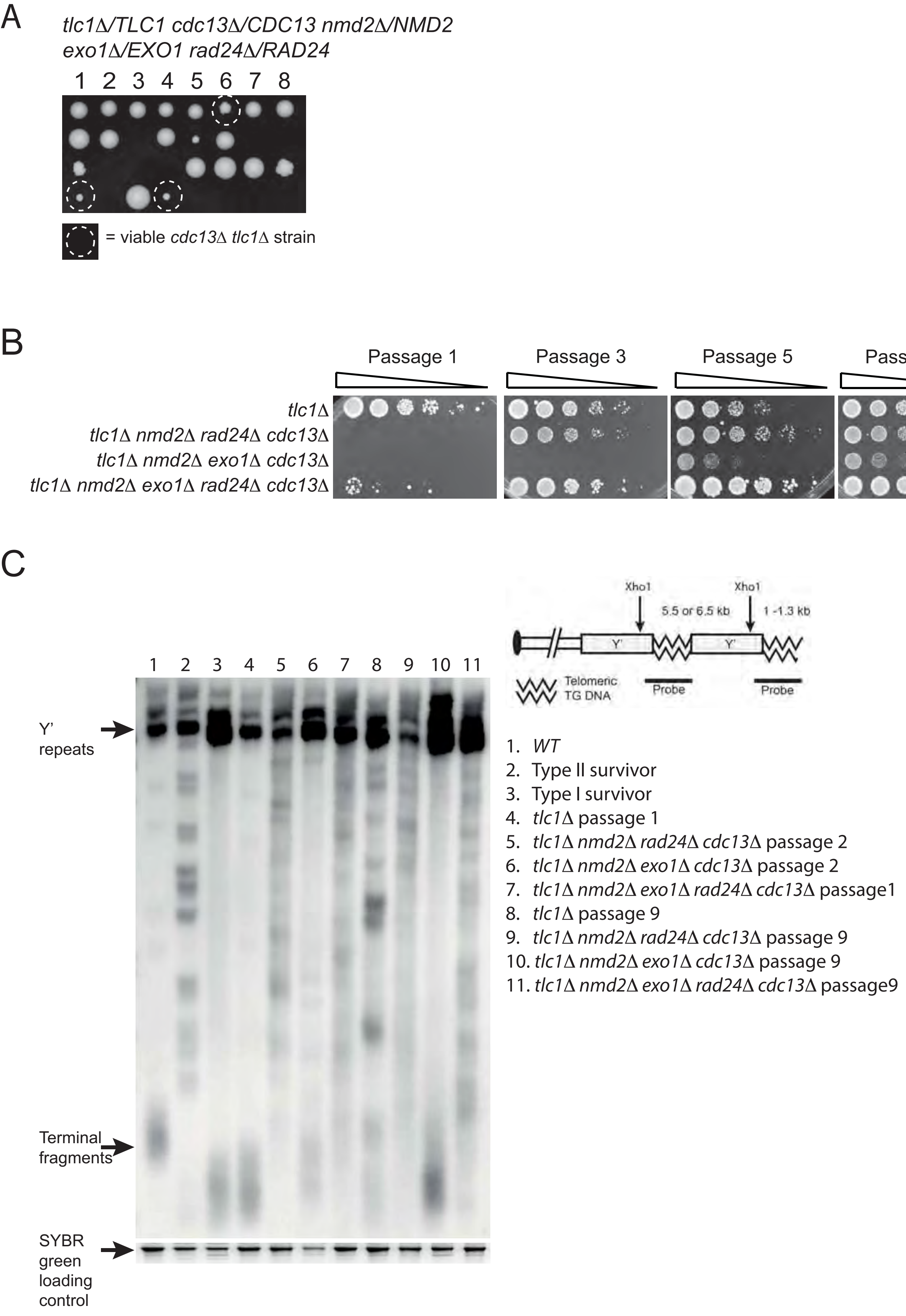
A
tlc1Δ/TLC1 cdc13Δ/CDC13 nmd2Δ/NMD2
exo1Δ/EXO1 rad24Δ/RAD24
1 2 3 4 5 6 7 8
= viable cdc13Δ tlc1Δ strain
B
Passage 1
Passage 3
Passage 5
Passage 7
tlc1Δ
tlc1Δ nmd2Δ rad24Δ cdc13Δ
tlc1Δ nmd2Δ exo1Δ cdc13Δ
tlc1Δ nmd2Δ exo1Δ rad24Δ cdc13Δ
C
Xho1
Xho1
5.5 or 6.5 kb
1 -1.3 kb
Y'
Y'
Telomeric TG DNA
Probe
Probe
1 2 3 4 5 6 7 8 9 10 11
Y' repeats
Terminal fragments
SYBR green loading control
1. WT
2. Type II survivor
3. Type I survivor
4. tlc1Δ passage 1
5. tlc1Δ nmd2Δ rad24Δ cdc13Δ passage 2
6. tlc1Δ nmd2Δ exo1Δ cdc13Δ passage 2
7. tlc1Δ nmd2Δ exo1Δ rad24Δ cdc13Δ passage1
8. tlc1Δ passage 9
9. tlc1Δ nmd2Δ rad24Δ cdc13Δ passage 9
10. tlc1Δ nmd2Δ exo1Δ cdc13Δ passage 9
11. tlc1Δ nmd2Δ exo1Δ rad24Δ cdc13Δ passage9

Figure S3. Viable *cdc13*Δ strains survive without telomerase. Related to Figure 3.

(A) Diploids containing heterozygous deletion mutations of *TLC1*, *EXO1, NMD2, RAD24* and *CDC13* were sporulated. Tetrads were dissected and germinated on YEPD plates. Spores were allowed to form colonies for 5 days at 23°C before being photographed. Genotypes were determined by growth on appropriate selective plates. (B) Strains of the genotypes indicated were repeatedly passaged by toothpick every 4 days at 23°C. At the indicated times 2 ml liquid cultures were grown overnight, serially diluted, spotted onto YEPD plates and incubated for 2 days before being photographed. (C) Genomic DNA was isolated from yeast strains indicated and telomere structures were analyzed by Southern blotting using a Y' and TG probe. SYBR Safe was used as a loading control.

Figure S4

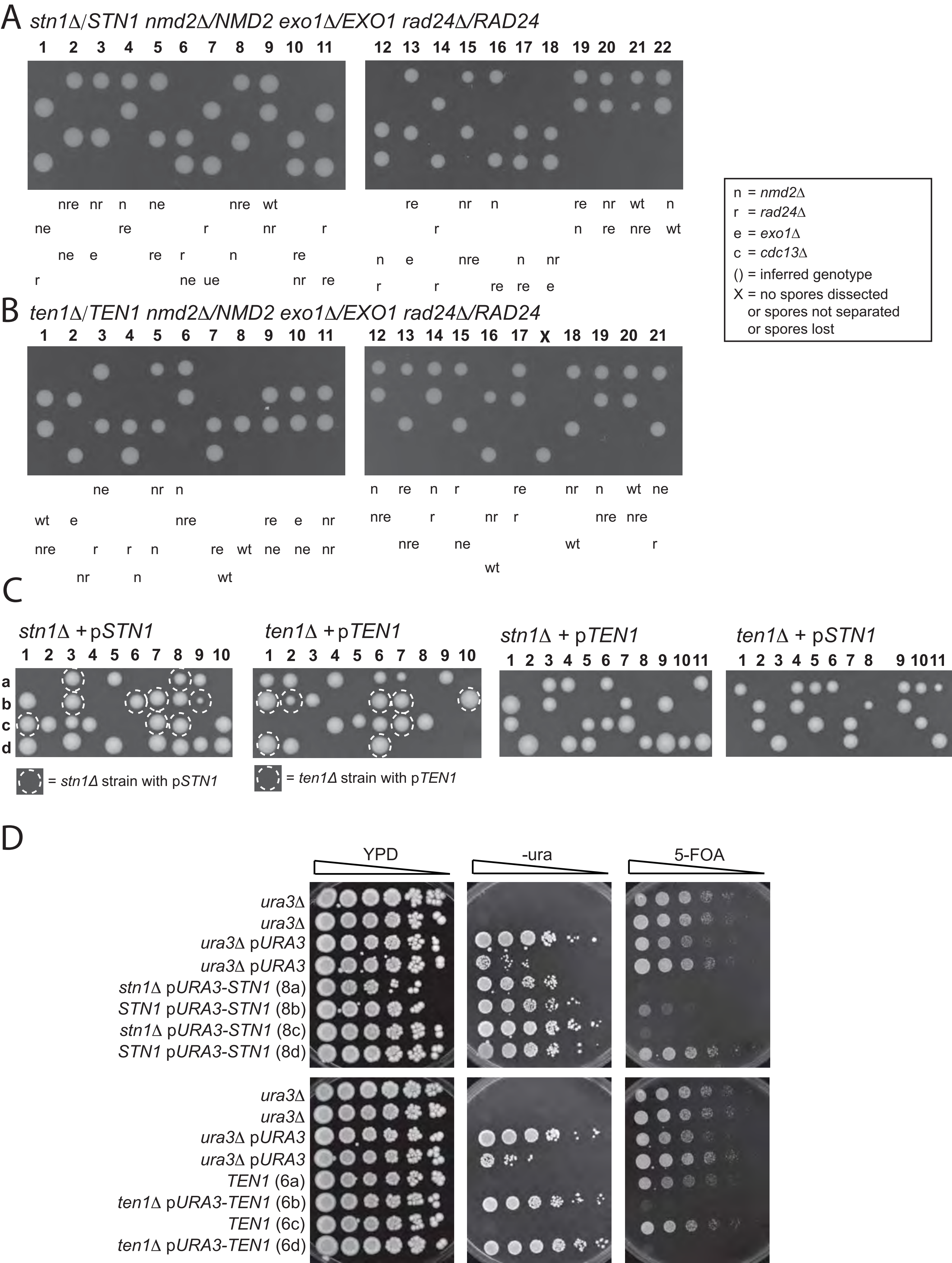
A stn1Δ/STN1 nmd2Δ/NMD2 exo1Δ/EXO1 rad24Δ/RAD24
B ten1Δ/TEN1 nmd2Δ/NMD2 exo1Δ/EXO1 rad24Δ/RAD24
n = nmd2Δ
r = rad24Δ
e = exo1Δ
c = cdc13Δ
() = inferred genotype
X = no spores dissected or spores not separated or spores lost
C
stn1Δ + pSTN1
ten1Δ + pTEN1
stn1Δ + pTEN1
ten1Δ + pSTN1
= stn1Δ strain with pSTN1
= ten1Δ strain with pTEN1
D
YPD
-ura
5-FOA
ura3Δ
ura3Δ
ura3Δ pURA3
ura3Δ pURA3
stn1Δ pURA3-STN1 (8a)
STN1 pURA3-STN1 (8b)
stn1Δ pURA3-STN1 (8c)
STN1 pURA3-STN1 (8d)
ura3Δ
ura3Δ
ura3Δ pURA3
ura3Δ pURA3
TEN1 (6a)
ten1Δ pURA3-TEN1 (6b)
TEN1 (6c)
ten1Δ pURA3-TEN1 (6d)

Figure S4. Growth of *stn1*Δ and *ten1*Δ strains. Related to Figure 4.

(A)-(B) Diploids containing heterozygous deletion mutations of *EXO1, NMD2, RAD24* along with deletions of *STN1* or *TEN1* were sporulated. Tetrads were dissected and germinated on YEPD plates. Spores were allowed to form colonies for 5 days at 23°C before being photographed. Genotypes were determined by growth on appropriate selective plates. (C) Diploid yeast strains in (A) and (B) were transformed with linearized plasmid and PCR fragments containing either *STN1* or *TEN1*, to obtain strains expressing *STN1* or *TEN1* on plasmids under their native promoters. Strains were treated as in (A)-(B). (D) Saturated cultures were serially diluted across agar plates containing YPD, synthetic medium without uracil (-ura) or synthetic medium with 5-fluoroorotic acid (5-FOA) and then allowed to form colonies at 30°C for 3 days before being photographed. Strains 8a-d in (D) were *STN1/stn1Δ* strains 8a-d in (C). Strains 6a-d in (B) were *TEN1/ten1Δ* strains 6a-d in (C).

Figure S5

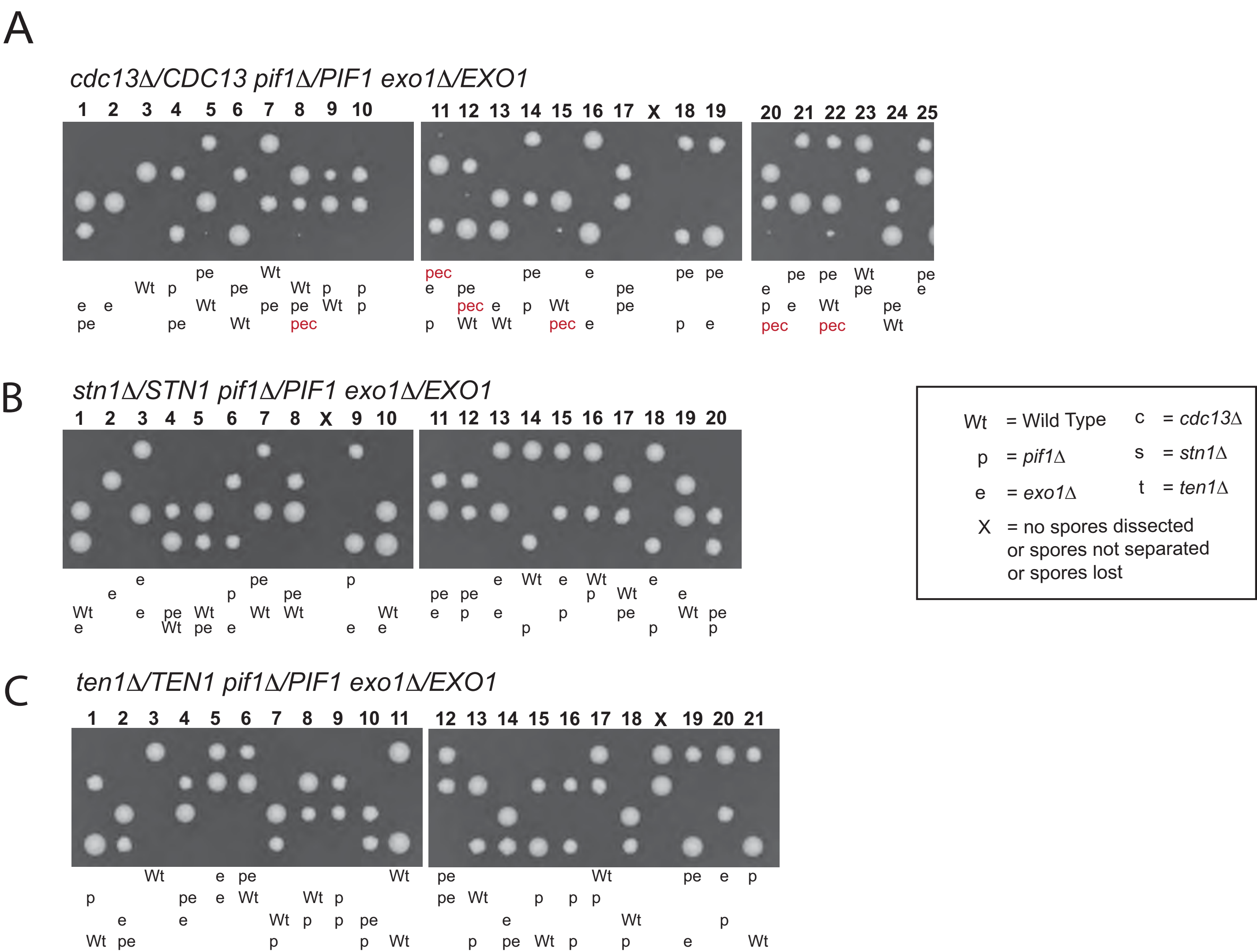

A
cdc13Δ/CDC13 pif1Δ/PIF1 exo1Δ/EXO1
1 2 3 4 5 6 7 8 9 10
11 12 13 14 15 16 17 X 18 19
20 21 22 23 24 25
pe Wt pec pe e pe pe pe pe pe Wt pe
Wt p pe Wt p p e pe pe e pe e
e e Wt pe pe Wt p pec e p Wt pe p e Wt pe
pe pe Wt pec p Wt Wt pec e p e pec pec Wt
B
stn1Δ/STN1 pif1Δ/PIF1 exo1Δ/EXO1
1 2 3 4 5 6 7 8 X 9 10
11 12 13 14 15 16 17 18 19 20
e pe p e Wt e Wt e
e p pe pe pe p Wt e
Wt e pe Wt Wt Wt Wt e p e p pe Wt pe
e Wt pe e e e p p p
C
ten1Δ/TEN1 pif1Δ/PIF1 exo1Δ/EXO1
1 2 3 4 5 6 7 8 9 10 11
12 13 14 15 16 17 18 X 19 20 21
Wt e pe Wt pe Wt pe e p
p pe e Wt Wt p pe Wt p p p
e e Wt p p pe e Wt p
Wt pe p p Wt p pe Wt p p e Wt
Wt = Wild Type
p = pif1Δ
e = exo1Δ
c = cdc13Δ
s = stn1Δ
t = ten1Δ
X = no spores dissected or spores not separated or spores lost

Figure S5. Bypass of *cdc13*Δ but not *stn1*Δ or *ten1*Δ by *pif1*Δ *exo1*Δ mutations. Related to Figure 5.

(A)-(C) Diploids containing heterozygous deletion mutations of *EXO1* and *PIF1*, along with deletions of *CDC13, STN1* or *TEN1* were sporulated. Tetrads were dissected and germinated on YEPD plates. Spores were allowed to form colonies for 5 days at 23°C before being photographed. Genotypes were determined by growth on appropriate selective plates.

Supplemental Experimental Procedures for

Interactions between CST, DDR and NMD genes show that Stn1 and Ten1 are the key components of the CST complex

Eva-Maria Holstein, Kate R. M. Clark and David Lydall
Correspondence to: David.Lydall@newcastle.ac.uk

This file includes:

1. List of strains used
2. List of primers used
3. List of plasmids used

1. List of yeast strains used. Related figures indicated.

Strain (DLY)	Relevant Genotype	Related Figures
640	*MAT*a	1B, 2G, S1A
1255	*MAT*a *cdc13-1 int rad9::HIS3*	2G, S1A
1272	*MAT*alpha *exo1::LEU2*	5D-E
1273	*MAT*alpha *exo1::LEU2*	5D-E
1296	*MAT*a *exo1::LEU2 cdc13-1 int*	1B, 2G, S1A
1385	*MAT*a *ura3* pRS415*::URA3*	S4D
1468	*MAT*a *cdc13-1 int cdc15-2 bar1::hisG*	2B-E, S1B
1470	*MAT*a *cdc13-1 int rad9::HIS3 cdc15-2 bar1::hisG*	2B-E, S1B
1522	*MAT*a *cdc13-1 int rad24::TRP1*	1B
1628	*MAT*alpha *tlc1::HIS3 pTLC1::URA3*	3C-D
1692	*MAT*a *exo1::LEU2 rad9::HIS3 cdc13-1 int*	2G S1A
1696	*MAT*alpha *cdc13-1 int exo1::LEU2 rad24::TRP1*	1B
2146	*MAT*a *tlc1 ::HIS3*	S3C
2148	*MAT*alpha *tlc1::HIS3 exo1::LEU2*	S3C
3001	*MAT*alpha	5D-E, S4D
4528	*MAT*a *nmd2::HIS3*	6F
4557	*MAT*a *cdc13-1 int*	1B, 2F-G, S1A
4625	*MAT*a *cdc13-1 int nmd2::HIS3*	1B, 2F-G, S1A
4765	*MAT*a *nmd2::HIS3*	1B, 2G, S1A
4766	*MAT*alpha *nmd2::HIS3*	5D-E
4872	*MAT*a *pif1::NATMX*	5D-E
4873	*MAT*alpha *pif1::NATMX*	5D-E
5255	*MAT*alpha *rad9::LEU2 nmd2::HIS3 cdc13-1 int*	2G, S1A
5673	*MAT*a *nmd2::HIS3 rad9::LEU2 cdc13-1 int cdc15-2 bar1::hisG*	2B-E, S1B
5674	*MAT*a *nmd2::HIS3 cdc13-1 int cdc15-2 bar1::hisG*	2B-E, S1B
5750	*MAT*alpha *ura3 pAG36::URA3*	S4D
5759	*MAT*a *STN1-C-MYC::TRP1 nmd2::HIS3*	6B
5761	*MAT*a *STN1-C-MYC::TRP1*	6B
5915	*MAT*a *pif1::NATMX*	5D-E
5916	*MAT*alpha *pif1::NATMX*	5D-E
7273	*MAT*alpha *CDC13-C-MYC::HIS3*	6A
7274	*MAT*a *CDC13-C-MYC::HIS3 nmd2::URA3*	6A
7747	*MAT*a *cdc13-1 int exo1::LEU2 nmd2::URA3*	1B
7749	*MAT*a *cdc13-1 int rad24::TRP1 nmd2::URA3*	1B
7752	*MAT*a *cdc13-1 int rad24::TRP1 exo1::LEU2 nmd2::URA3*	1B
8100	*MAT*a *nmd2::URA3 TEN1-C-MYC::KANMX*	6C
8101	*MAT*a *TEN1-C-MYC::KANMX*	6C
8167	*nmd2::HIS3 exo1::LEU2 cdc13::HPHMX* passage	3C-D

	1	
8168	*nmd2::HIS3 exo1::LEU2 cdc13::HPHMX* passage 1	3D
8169	*nmd2::HIS3 exo1::LEU2 rad24::TRP1 cdc13::HPHMX* passage 1	3C-D
8170	*nmd2::HIS3 exo1::LEU2 rad24::TRP1 cdc13::HPHMX* passage 1	3D
8171	*nmd2::HIS3 rad24::TRP1 cdc13::HPHMX* passage 1	3C-D
8172	*nmd2::HIS3 rad24::TRP1 cdc13::HPHMX* passage 1	3D
8198	*nmd2::HIS3 exo1::LEU2 rad24::TRP1*	6F
8199	*nmd2::HIS3 exo1::LEU2 rad24::TRP1 cdc13::HPHMX* passage 9	3C-D
8200	*nmd2::HIS3 exo1::LEU2 rad24::TRP1 cdc13::HPHMX* passage 9	3D
8201	*nmd2::HIS3 rad24::TRP1 cdc13::HPHMX* passage 9	3C-D
8202	*nmd2::HIS3 rad24::TRP1 cdc13::HPHMX* passage 9	3D
8203	*nmd2::HIS3 exo1::LEU2 cdc13::HPHMX* passage 9	3C-D
8204	*nmd2::HIS3 exo1::LEU2 cdc13::HPHMX* passage 9	3D
8432	*MAT*a *STN1-13MYC::KANMX nmd2::HIS3 exo1::LEU2 rad24::TRP1* passage 8	6D
8434	*MAT*alpha *TEN1-13MYC::KANMX nmd2::HIS3 exo1::LEU2 rad24::TRP1* passage 8	6E
8460	*MAT*a	3C, S3A, 6F
8461	*nmd2::HIS3 exo1::LEU2 rad24::TRP1 cdc13::HPHMX*	6F
8462	*MAT*a *tlc1::NATMX* passage 1	S3B-C
8463	*MAT*a *tlc1::NATMX nmd2::HIS3 rad24::TRP1 cdc13::HPHMX* passage 1	S3B
8464	*MAT*a *tlc1::NATMX nmd2::HIS3 exo1::LEU2 cdc13::HPHMX* passage 1	S3B
8465	*MAT*a *tlc1::NATMX nmd2::HIS3 exo1::LEU2 rad24::TRP1 cdc13::HPHMX* passage 1	S3B-C
8467	*MAT*a *tlc1::NATMX nmd2::HIS3 rad24::TRP1 cdc13::HPHMX* passage 2	S3C
8468	*MAT*a *tlc1::NATMX nmd2::HIS3 exo1::LEU2 cdc13::HPHMX* passage 2	S3C
8472	*MAT*a *tlc1::NATMX* passage 3	S3B
8473	*MAT*a *tlc1::NATMX nmd2::HIS3 rad24::TRP1 cdc13::HPHMX* passage 3	S3B
8474	*MAT*a *tlc1::NATMX nmd2::HIS3 exo1::LEU2 cdc13::HPHMX* passage 3	S3B

8475	*MAT*a *tlc1::NATMX nmd2::HIS3 exo1::LEU2 rad24::TRP1 cdc13::HPHMX* passage 3	S3B
8509	*MAT*a *tlc1::NATMX* passage 5	S3B
8510	*MAT*a *tlc1::NATMX nmd2::HIS3 rad24::TRP1 cdc13::HPHMX* passage 5	S3B
8511	*MAT*a *tlc1::NATMX nmd2::HIS3 exo1::LEU2 cdc13::HPHMX* passage 5	S3B
8512	*MAT*a *tlc1::NATMX nmd2::HIS3 exo1::LEU2 rad24::TRP1 cdc13::HPHMX* passage 5	S3B
8551	*MAT*a *tlc1::NATMX* passage 7	S3B
8552	*MAT*a *tlc1::NATMX nmd2::HIS3 rad24::TRP1 cdc13::hph* passage 7	S3B
8553	*MAT*a *tlc1::NATMX nmd2::HIS3 exo1::LEU2 cdc13::hph* passage 7	S3B
8554	*MAT*a *tlc1::NATMX nmd2::HIS3 exo1::LEU2 rad24::TRP1 cdc13::hph* passage 7	S3B
8600	*MAT*a *tlc1::NATMX* passage 9	S3C
8601	*MAT*a *tlc1::NATMX nmd2::HIS3 rad24::TRP1 cdc13::HPHMX* passage 9	S3C
8602	*MAT*a *tlc1::NATMX nmd2::HIS3 exo1::LEU2 cdc13::hph* passage 9	S3C
8603	*MAT*a *tlc1::NATMX nmd2::HIS3 exo1::LEU2 rad24::TRP1 cdc13::HPHMX* passage 9	S3C
8604	*MAT*alpha *STN1::13MYC::KANMX nmd2::HIS3 exo1::LEU2 rad24::TRP1 cdc13::HPHMX* passage 8	5D
8605	*MAT*a *TEN1::13Myc::KANMX nmd2::HIS3 exo1::LEU2 rad24::TRP1 cdc13::HPHMX* passage 8	5E
**Strain (DDY)**	**Diploid Genotype**	**RELATED FIGURES**
340	*PIF1/pif1::NATMX EXO1/exo1::LEU2 CDC13/cdc13::HPHMX*	5A, S5A
355	*PIF1/pif1::NATMX EXO1/exo1::LEU2 STN1/stn1::HPHMX*	5B, S5B
566	*NMD2/nmd2::HIS3 EXO1/exo1::LEU2 RAD24/rad24::TRP1*	-
568	*NMD2/nmd2::HIS3 EXO1/exo1::LEU2 RAD24/rad24::TRP1 CDC13/cdc13::hph* (DDY 566 transformation)	3A-B, 6D-E,
569	*NMD2/nmd2::HIS3 EXO1/exo1::LEU2 RAD24/rad24::TRP1 STN1/stn1::hph* (DDY 566 transformation)	4A, S4A
571	*NMD2/nmd2::HIS3 EXO1/exo1::LEU2 RAD24/rad24::TRP1 TEN1/ten1::hph* (DDY 566 transformation)	4B
572	*NMD2/nmd2::HIS3 EXO1/exo1::LEU2 RAD24/rad24::TRP1 TEN1/ten1::hph* (DDY 566	4B, S4B

	transformation)	
573	*PIF1/pif1::NATMX EXO1/exo1::LEU2 TEN1/ten1::HPHMX*	5C, S5C
581	*NMD2/nmd2::HIS3 EXO1/exo1::LEU2 RAD24/rad24::TRP1 CDC13/cdc13::HPHMX TLC1/tlc1::NATMX* (DDY568 transformation)	S3A

2. List of oligonucleotides used. Related figures indicated.

Oligo Number	Primer Sequence	Target	Comments (Related Figures)
M417	ATGGGCGCAGGGCCTGGAT TC	Tag for QAOS	Tag/QAOS (Figure 2)
M418	ATGCTCGCAGAGCCCCTGG ATCT	Tag for QAOS	Tag/QAOS (Figure 2)
M1003	AGAGATGCGCCGTTATTGA CGATGCGAGTTTTCTAACAA Agaattcgagctcgtttaaac	*STN1*	For C-terminal Myc-tagging of *STN1* (Figure 6)
M1172	cagaccgaactcggtgattt	*BUD6*	Forward primer qRT-PCR (Figure 5)
M1173	ttttagcgggctgagaccta	*BUD6*	Reverse primer qRT-PCR (Figure 5)
M1509	CAAGAAATGGGAACCTCCA TTAAGATTAGCAA	*RET2*	Reverse primer/QAOS (Figure 2)
M1510	AGCGCACCTGCATCGTTGG CAGCAA	*RET2*	Probe/QAOS (Figure 2)
M1511	ATGGGCGCAGGGCCTGGAT TCATATGGAGCTT	*RET2*	Tagging primer/QAOS (Figure 2)
M1514	TGACTTTCCAAGATGCCTTG AACACTAGTGTC	*DUG1*	Reverse primer/QAOS (Figure 2)
M1515	AATGAGCACCATCATCGCC TCTAC	*DUG1*	Probe/QAOS (Figure 2)
M1516	ATGCTCGCAGAGCCCCTGG ATCTTCATACCACCA	*DUG1*	Tagging primer/QAOS (Figure 2)
M1734	TCGAGCAACTGCAAGAAGA A	*STN1*	Forward primer qRT-PCR (Figure 5)
M1735	CGAAATGACAAGGAATGCA C	*STN1*	Reverse primer qRT-PCR (Figure 5)
M1794	ATACACCAAAGTCCGCCAA T	*TEN1*	Forward primer qRT-PCR (Figure 5)

M1795	CACCAAGTGGTGATTTGAC A	*TEN1*	Reverse primer qRT-PCR (Figure 5)
M1803	CCAACGTCAACGCACC	*TLC1*	To confirm integration of *NATMX* cassette (Figure S3)
M1807	CGTGAGTCTGTGGAATCC	*TLC1*	To confirm integration of *NATMX* cassette (Figure S3)
M1828	CTCTTTGGATACGAATGACC GTGGAAACTATCGCCTAAA ACACATACGATTTAGGTGA CAC	*CDC13*	To integrate hph cassette at *CDC13* locus: M1828, M1829 (Figure 3)
M1829	GCAATTTGGCACCGCCGCG TTGGGCTGCGCGGATCATGT CAATACGACTCACTATAGG GAG	*CDC13*	To integrate hph cassette at *CDC13* locus: M1828, M1829 (Figure 3)
M1897	TCTCCGCCATAGAATCATCA	*CDC13*	To confirm integration: M1897, M1898 (Figure 3)
M1898	GCGTTAAACGTAAATACCT CTTTC	*CDC13*	To confirm integration: M1897, M1898 (Figure 3)
M1943	GCGAAAAAAGAGCAATAAG CTGGCTTTTAATAATGGGTT GGAGCCCACATACGATTTA GGTGACAC	*STN1*	To integrate hph cassette at *STN1* locus: M1943, M1944 (Figure 3)
M1944	CCTAGAGAGATGCGCCGTT ATTGACGATGCGAGTTTTCT AACAAAAATACGACTCACT ATAGGGAG	*STN1*	To integrate hph cassette at *STN1* locus: M1943, M1944 (Figure 3)

M2188	[CY5] CCCACCACACACACCCACACCC	*TG ssDNA*	Probe/ Fluorescent in-gel assay (Figure 2)
M1367	AATAACGAATTGAGCTATGACACCAA	*PAC2*	ChIP (Figure 5)
M1368	AGCTTACTCATATCGATTTCATACGACTT	*PAC2*	ChIP (Figure 5)
M2013	CAATTAAAAGACCTTCTTTGTAGCTTTTAGTGTGATTTTTCTGGTTTGAGCACATACGATTTAGGTGACAC	*TLC1*	To integrate *NATMX* cassette at *TLC1* locus (Figure S3)
M2014	TGTATATTGTATATTCTAAAAAGAAGAAGCCATTTGGTGGGCTTTATTA AATACGACTCACTATAGGGAG	*TLC1*	To integrate *NATMX* cassette at *TLC1* locus (Figure S3)
M2245	CGTATGCTAAAGTATATATTACTTCACTCCATT	chrVI-R	ChIP (Figure 4)
M2246	TCCGAACTCAGTTACTATTGATGGAA	chrVI-R	ChIP (Figure 4)
M2355	ATCACCACTTGGTGAGAAGTTTCTGAACATCTCTAACTCCCGGATCCCCGGGTTAATTAA	*TEN1*	For C-terminal Myc-tagging of *TEN1* (Figure 6)
M2356	AAAGGTATTATATAATCTCAGTATATGCCAAACGTCTGACGAATTCGAGCTCGTTTAAAC	*TEN1*	For C-terminal Myc-tagging of *STN1* (Figure 6)
M2667	AGCTCTATTTGCTCTTTTTAGAGAACTACGACAGTGTTATCACATACGATTTAGGTGACAC	*TEN1*	To integrate *HPHMX* cassette at *TEN1* locus: M2667, M2668 (Figure 3)
M2668	TATCAAAGGTATTATATAATCTCAGTATATGCCAAACGTCTGACAATACGACTCACTATAGGGAG	*TEN1*	To integrate *HPHMX* cassette at *TEN1* locus: M2667, M2668 (Figure 3)
M2669	CAGTTGTCGTTATTTTTCTTTT	*TEN1*	To confirm *HPHMX* integration: M2669, M2670 (Figure 3)

M2670	CTTTCCTTTCTTTTCTCATAC TTATT	*TEN1*	To confirm *HPHMX* integration: M2669, M2670 (Figure 3)
M2671	CTGCTTGTTGATATTCCATT T	*STN1*	To confirm *HPHMX* integration: M2671, M2672 (Figure 3)
M2672	AATTTTTCAGGCCTAGAGA GAT	*STN1*	To confirm *HPHMX* integration: M2671, M2672 (Figure 3)
M2735	ACCGCGGTGGCGGCCGCAT AGGCCACTAGTGGATCTGA TATCATCGATGCGATGCTCC GCAGGAATTCT	*STN1*	Amplify and clone into pDL1466: M2735, M2736 (Figure S3)
M2736	CAATTTATAAAGACTGGAG GCAATCACAAACCTCCTGCT CGACGGATAGCCTCTGTCTC TTCTTTCA	*STN1*	Amplify and clone into pDL1466: M2735, M2736 (Figure S3)
M2737	ACCGCGGTGGCGGCCGCAT AGGCCACTAGTGGATCTGA TATCATCGATGTACCCAAAC ATATAAATGCC	*TEN1*	Amplify and clone into pDL1466: M2737, M2739 (Figure S3)
M2739	CAATTTATAAAGACTGGAG GCAATCACAAACCTCCTGCT CGACGGATATCTAACCCAG AATTATTGA	*TEN1*	Amplify and clone into pDL1466: M2737, M2739 (Figure S3)
M2752	TGGAATATTATTACATTTGG AATACGCAGCAGCATATTC ACGGATCCCCGGGTTAATT AA	*STN1*	For C-terminal Myc-tagging of *STN1*

3. List of plasmids used. Related figures indicated.

Plasmid Number	Details	Source/Alias (Related Figures)
pDL508	kanMX cassette for C-terminal myc-tagging	pFA6a-13Myc-kanMX6. Longtine et al, Yeast 1998 14:953-961. (Figure 6)
pDL987	Cut with *Xho*I and *BamH*I and use 1000bp fragment to detect Y' and telomeric repeat sequences.	pHT128 (pYTEL). Tsubouchi and Ogawa. MBOC. 2000 11:2221 2233 (Figure 3D, Figure S3C)
pDL1389	Plasmid containing hphMX4	pAG32 (Figures 3-4, S3-4)
pDL1221	Plasmid containing natMX4	pAG25 (Figure S3)
pDL1466	Plasmid containing *URA3* and *CAN1*. Cut with *Sph*I and *Asc*I for PCR-mediated gap repair.	Created by Marion Dubarry (Newcastle University); derivative of pDL1279 (Figure S3)
