# Supplementary material for: Interplay between Nonsense-Mediated mRNA Decay and DNA Damage Response Pathways Reveals that Stn1 and Ten1 Are the Key CST Telomere-Cap Components
Source: Cell Rep. 2014 May 15;7(4):1259–69. doi: 10.1016/j.celrep.2014.04.017 (PMC4518466; doi:10.1016/j.celrep.2014.04.017)
Supplement: Document S2. Article plus Supplemental Information [file mmc2.pdf]

OPEN ACCESS
CellPress

Interplay between Nonsense-Mediated mRNA Decay and DNA Damage Response Pathways Reveals that Stn1 and Ten1 Are the Key CST Telomere-Cap Components

Eva-Maria Holstein,[1,2] Kate R.M. Clark,[1,2] and David Lydall[1,*]
[1]Institute for Cell and Molecular Biosciences, Newcastle University Medical School, Newcastle upon Tyne NE2 4HH, UK
[2]Co-first author
*Correspondence: david.lydall@newcastle.ac.uk
http://dx.doi.org/10.1016/j.celrep.2014.04.017
This is an open access article under the CC BY license (http://creativecommons.org/licenses/by/3.0/).

SUMMARY

A large and diverse set of proteins, including CST complex, nonsense mediated decay (NMD), and DNA damage response (DDR) proteins, play important roles at the telomere in mammals and yeast. Here, we report that NMD, like the DDR, affects single-stranded DNA (ssDNA) production at uncapped telomeres. Remarkably, we find that the requirement for Cdc13, one of the components of CST, can be efficiently bypassed when aspects of DDR and NMD pathways are inactivated. However, identical genetic interventions do not bypass the need for Stn1 and Ten1, the partners of Cdc13. We show that disabling NMD alters the stoichiometry of CST components at telomeres and permits Stn1 to bind telomeres in the absence of Cdc13. Our data support a model that Stn1 and Ten1 can function in a Cdc13-independent manner and have implications for the function of CST components across eukaryotes.

INTRODUCTION

Telomeres are complex nucleoprotein structures that protect chromosome ends from DNA damage responses (DDR). The most terminal DNA on a chromosome is typically G-rich 3′ single-stranded DNA (ssDNA), resembling a DNA double-strand break (DSB) in the process of repair by homologous recombination. In budding yeast, CST (Cdc13, Stn1, and Ten1), proteins are proposed to form a heterotrimeric telomeric ssDNA-binding complex, that helps cap telomeres and is analogous to the heterotrimeric RPA complex that binds nuclear ssDNA during the process of transcription, DNA replication, and repair (Gao et al., 2007; Sun et al., 2009, 2011). Cdc13 binds telomeric ssDNA strongly via an oligonucleotide/oligosacccharide binding (OB) fold (Lewis et al., 2014). Stn1 and Ten1 also bind telomeric ssDNA but with lower affinity than Cdc13 and are thought to be recruited to DNA via Cdc13 (Gao et al., 2007; Qian et al., 2009, 2010). So far, the budding yeast CST complex has not been purified, but recent evidence from the distant yeast *Candida glabrata* suggests that in this organism CST functions as a 2:4:2 or 2:6:2 complex (Lue et al., 2013).

Orthologs of CST components have recently been identified in mammals, plants, and fission yeast. The human components of CST (CTC1, STN1 [OBFC1], and TEN1) can be purified as a trimeric complex (Chen et al., 2012; Giraud-Panis et al., 2010; Miyake et al., 2009; Surovtseva et al., 2009). Mutations in CTC1 are associated with human diseases and have been associated with cellular telomere defects (Chen et al., 2013; Anderson et al., 2012). Interestingly, CTC1 and STN1 were originally identified when copurified with human DNA polymerase alpha and named alpha accessory factor (AAF) (Casteel et al., 2009). The interaction of CST with DNA polymerase alpha is conserved because budding yeast Cdc13 and Stn1 also bind to DNA polymerase alpha components (Qi and Zakian, 2000; Grossi et al., 2004).

In budding yeast, where CST was first identified, there is evidence that CST subunits perform different functions. For example, Cdc13 helps recruit telomerase via interaction with the telomerase subunit Est1 (Nugent et al., 1996; Qi and Zakian, 2000; Mitton-Fry et al., 2004). In contrast, Stn1 interferes with telomerase activity because Stn1 and Est1 have overlapping binding sites on Cdc13, and Stn1 inhibits telomerase activity by competing with Est1 for Cdc13 binding (Puglisi et al., 2008; Chandra et al., 2001). Another example is that Stn1, when overproduced, acts as a checkpoint inhibitor (Gasparyan et al., 2009). However, because Cdc13, Stn1, and Ten1 are each essential proteins in budding yeast, and there is clear homology to RPA, it is suggested that CST proteins function together to provide the essential function of capping the telomere (Gao et al., 2007).

In yeast and human cells, nonsense mediated mRNA decay (NMD) proteins play important roles at telomeres. NMD degrades transcripts containing premature termination codons (PTCs) to reduce the risk that potentially harmful truncated proteins (or RNA) are made in cells (Isken and Maquat, 2008). It is estimated that about 10% of human diseases are associated with PTCs (Bidou et al., 2012). In human cells, the key NMD proteins UPF1, UPF2, and UPF3 bind to telomeres, and telomere loss occurs in UPF1 and UPF2-depleted cells (Lew et al., 1998; Azzalin et al., 2007). Consistent with the telomere effect in human cells, budding yeast *nmd*Δ mutants show a short telomere phenotype. Interestingly, in yeast *nmd*Δ mutants overexpression

CrossMark

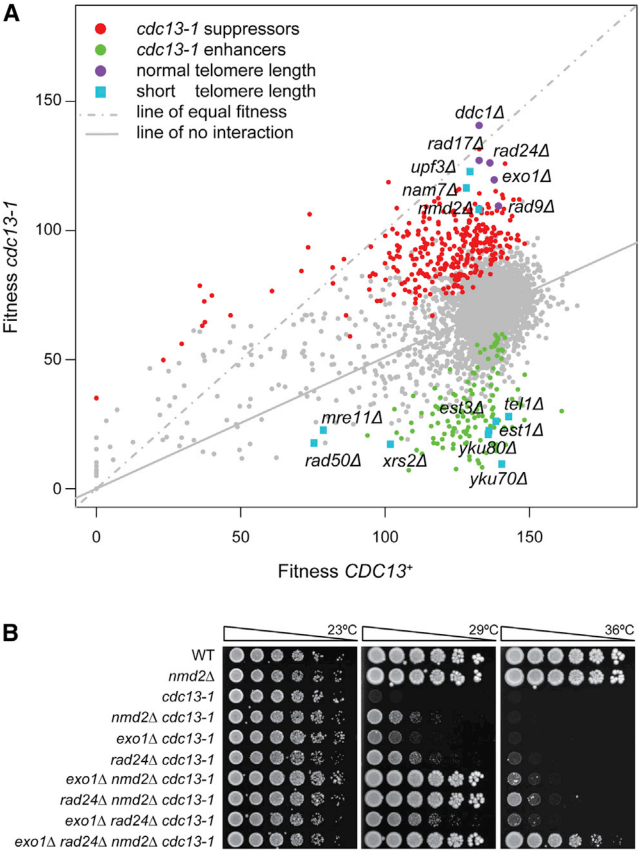

Figure 1. Deletion Mutations that Suppress or Enhance *cdc13-1*

(A) *cdc13-1* or *CDC13* strains were combined with the yeast knockout collection and fitness (maximum doubling rate × maximum doubling potential) determined at 27°C (Addinall et al., 2011). Each spot corresponds to the position of a single gene deletion. *cdc13-1* suppressors (red) or enhancers (green) are indicated, as are deletions known to affect telomere length (blue) or the DNA damage response (purple).

(B) Saturated cultures, grown at 23°C, were serially diluted in water and spotted onto YEPD plates. Strains were grown at the temperatures indicated for 2 days before being photographed.

of Stn1 and Ten1 is largely responsible for the short telomere length phenotype (Dahlseid et al., 2003). This is presumably because Stn1/Ten1 inhibits telomerase activity by interfering with Est1-Cdc13 interaction.

We have previously reported that disabling NMD (*NAM7*, *NMD2*, and *UPF3*) or DDR genes such as *EXO1,* encoding a nuclease, or *RAD24*, encoding the checkpoint sliding clamp loader, suppresses temperature sensitivity of telomere-defective *cdc13-1* strains to similar extents (Addinall et al., 2011). Given the important roles played by CST, NMD, and DDR proteins at mammalian and yeast telomeres, we wanted to better understand the interplay between NMD and DDR at uncapped telomeres. Remarkably, we find that deleting *NMD2* with either *EXO1* or *RAD24* completely bypasses the requirement for Cdc13. However, the same genetic interventions do not bypass the need for either Stn1 or Ten1. These and other molecular experiments indicate that CST does not always function as an RPA-like trimeric protein in yeast. Instead, our data show that Stn1 and Ten1 are critical for cell viability in conditions when Cdc13 is not, and this suggests that Stn1 and Ten1 can cap telomeres, or perform other essential functions, in the absence Cdc13.

RESULTS

cdc13-1 Can Be Strongly Suppressed by *nmd2Δ* with *exo1Δ* and/or *rad24Δ*

The Cdc13-1 protein becomes increasingly defective at capping the telomere as temperatures increase. At high temperatures, *cdc13-1* cells accumulate telomeric ssDNA, activate checkpoint pathways, and arrest before anaphase (Garvik et al., 1995). To begin to systematically define the proteins and pathways that are important for telomere function, *cdc13-1* was combined with the yeast genome knockout collection to identify suppressors and enhancers of the temperature-sensitive telomere defect (Addinall et al., 2011). We found that deletions of NMD genes (*nam7Δ*, *nmd2Δ*, and *upf3Δ*), which cause short telomere phenotypes, suppress the *cdc13-1* defect strongly. The effects of *nmdΔ* mutations were as strong as deletions affecting aspects of the DNA Damage Response (DDR), including deletions of DNA damage checkpoint genes (*ddc1Δ*, *rad9Δ*, *rad17Δ*, and *rad24Δ*) or *exo1Δ*, affecting a nuclease that attacks uncapped telomeres (Figure 1A). Interestingly, other deletions affecting the DDR or telomerase cause a short telomere phenotype but enhanced the *cdc13-1* defect; such proteins include the Ku complex (Yku70, Yku80), the MRX complex (Mre11, Rad50, Xrs2), or telomerase (Est1 and Est3 regulatory subunits). Therefore, *nmdΔ* mutations are somewhat unusual in that they result in short telomeres but suppress *cdc13-1*.

To better understand the role of NMD at telomeres, we investigated the overlap between NMD and the DDR. We generated all possible combinations of *nmd2Δ*, *exo1Δ*, and *rad24Δ* mutations in *cdc13-1* strains. We observed strong synergistic interactions between *nmd2Δ* and *exo1Δ* or *rad24Δ* mutations. Specifically, deleting *nmd2Δ* in combination with *exo1Δ* or *rad24Δ* in *cdc13-1* strains significantly increased strain fitness compared to each single gene deletion (Figure 1B). In contrast, *exo1Δ rad24Δ* double deletions only marginally improved growth compared to *exo1Δ* or *rad24Δ* single deletions. We conclude that NMD inhibits the growth of *cdc13-1* mutants by a mechanism that is distinct to the effects of Exo1 and Rad24, which are more similar in effect. The *nmd2Δ rad24Δ exo1Δ cdc13-1* strain was most fit, growing robustly at 36°C, demonstrating that Nmd2, Rad24, and Exo1 each perform different functions to inhibit growth of *cdc13-1* mutants. The synergistic genetic interactions between NMD and the DDR indicate that that NMD functions in parallel to the DDR proteins Exo1 and Rad24 to inhibit growth of *cdc13-1* mutants.

nmd2Δ Affects ssDNA Accumulation in *cdc13-1* Strains

Exo1 and Rad24 inhibit growth of *cdc13-1* strains at least in part by generating single-stranded DNA (ssDNA) at uncapped telomeres (Zubko et al., 2004). To test the effect of Nmd2 on ssDNA, we measured ssDNA near telomeres in *nmd2Δ cdc13-1* and *nmd2Δ rad9Δ cdc13-1* strains. The checkpoint protein Rad9,

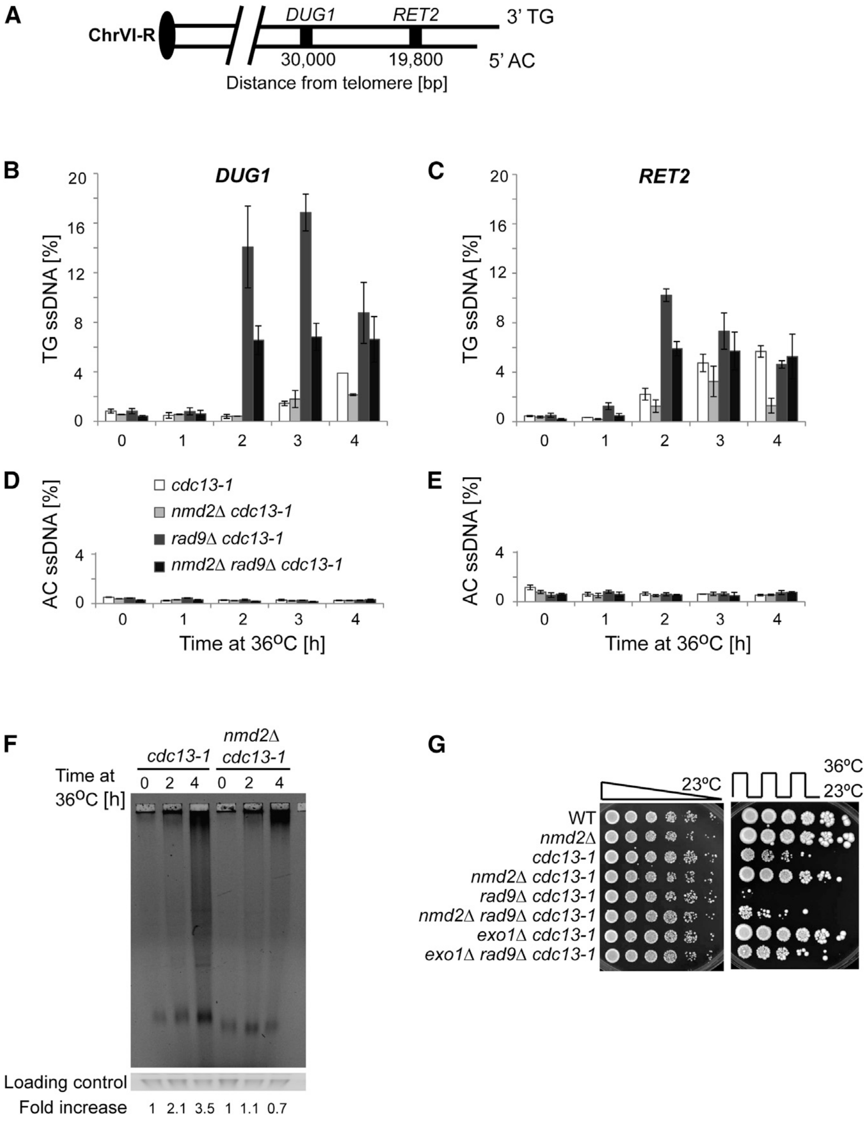

Figure 2. Nmd2 Affects ssDNA Generation at Uncapped Telomeres

(A) Schematic representation of chromosome arm VI-R.

(B–E) Quantitative amplification of single-stranded DNA (QAOS) isolated from synchronous cultures of cells containing *bar1Δ*, *cdc13-1*, *cdc15-2*, and other mutations indicated. Error bars show 95% confidence interval values.

(F) Cells dividing exponentially at 23°C were incubated at 36°C and ssDNA in the telomeric repeats was measured. SYBR Safe was used as a loading control. ssDNA was quantified using ImageJ and normalized relative to the loading control. The final fold change is relative to the 0 hr time point of each strain.

(G) Yeast strains indicated were grown to saturation at 23°C before being spotted on two plates. One plate was incubated at 23°C for 3 days, the other plate was incubated for three 4 hr cycles at 36°C, separated by 4 hr at 23°C, before colonies were allowed to form at 23°C.

See also Figure S1.

like its mammalian ortholog 53BP1, inhibits ssDNA accumulation and was used to sensitize some strains to the accumulation of ssDNA (Lazzaro et al., 2008; Bunting et al., 2010). We used quantitative amplification of single-stranded DNA (QAOS) to measure ssDNA accumulation at the *DUG1* and *RET2* loci on the right arm of chromosome VI-R (Holstein and Lydall, 2012) (Figure 2A). Deleting *NMD2* reduced the amount of ssDNA generated in *cdc13-1* or *cdc13-1 rad9Δ*-strains at loci 20 or 30 kb from uncapped telomeres (Figures 2B–2E). We further investigated the effect of deleting *NMD2* on telomeric ssDNA by using a fluorescent native in-gel assay to measure ssDNA in telomeric repeats in *nmd2Δ cdc13-1* strains, grown at a restrictive temperature. Consistent with the QAOS data, we observed reduced ssDNA accumulation in the telomeric repeats of *nmd2Δ cdc13-1* strains after 4 hr at 36°C (Figure 2F). To obtain independent evidence that *NMD2* affects ssDNA, we measured the effect of *nmd2Δ* on cell viability of *cdc13-1* and *cdc13-1 rad9Δ* strains subjected to restrictive and permissive temperature cycles in an "up-down" assay (Figures 2G and S1A). Deleting *NMD2* in a *cdc13-1* or *cdc13-1 rad9Δ* background increased cell viability assessed by spot tests after growth at 36°C, similar to the effect of deleting *EXO1* in the same backgrounds (Figure 2G). This spot test result was confirmed by determining cell viability after incubation at restrictive temperature: *nmd2Δ cdc13-1 rad9Δ* cultures contained nearly 8% viable cells compared to around 1% of *cdc13-1 rad9Δ* cultures at the 240 min time point (Figure S1B). We conclude that Nmd2, like Rad24 and Exo1, affects ssDNA levels in *cdc13-1* mutants.

nmd2Δ rescued loss of viability caused by rapid accumulation of ssDNA in *cdc13-1* mutants, similar to the previously reported effects of *exo1Δ* and *rad24Δ* mutations (Zubko et al., 2004). It is known that disabling NMD pathways increases the levels of many telomere related proteins and RNAs, including the Ku complex, telomerase, Telomeric Repeat Containing RNA (TERRA), and the Cdc13 partner proteins Stn1 and Ten1 (Guan et al., 2006; Azzalin et al., 2007; Dahlseid et al., 2003; Addinall et al., 2011). It is likely therefore that disabling the NMD pathway increases the levels of one or more of these telomere related proteins or RNAs and thereby reduces resection of telomeric ssDNA. Alternatively, NMD may regulate an unidentified nuclease that attacks telomeric DNA, play a direct role in resection, or affect the stability of ssDNA generated in *cdc13-1* strains.

The Requirement for *CDC13* Can Be Bypassed

The robust growth of *nmd2Δ rad24Δ exo1Δ cdc13-1* mutants at 36°C suggested that cells deficient in NMD and DDR might be able to divide in the absence of any Cdc13 function. To test

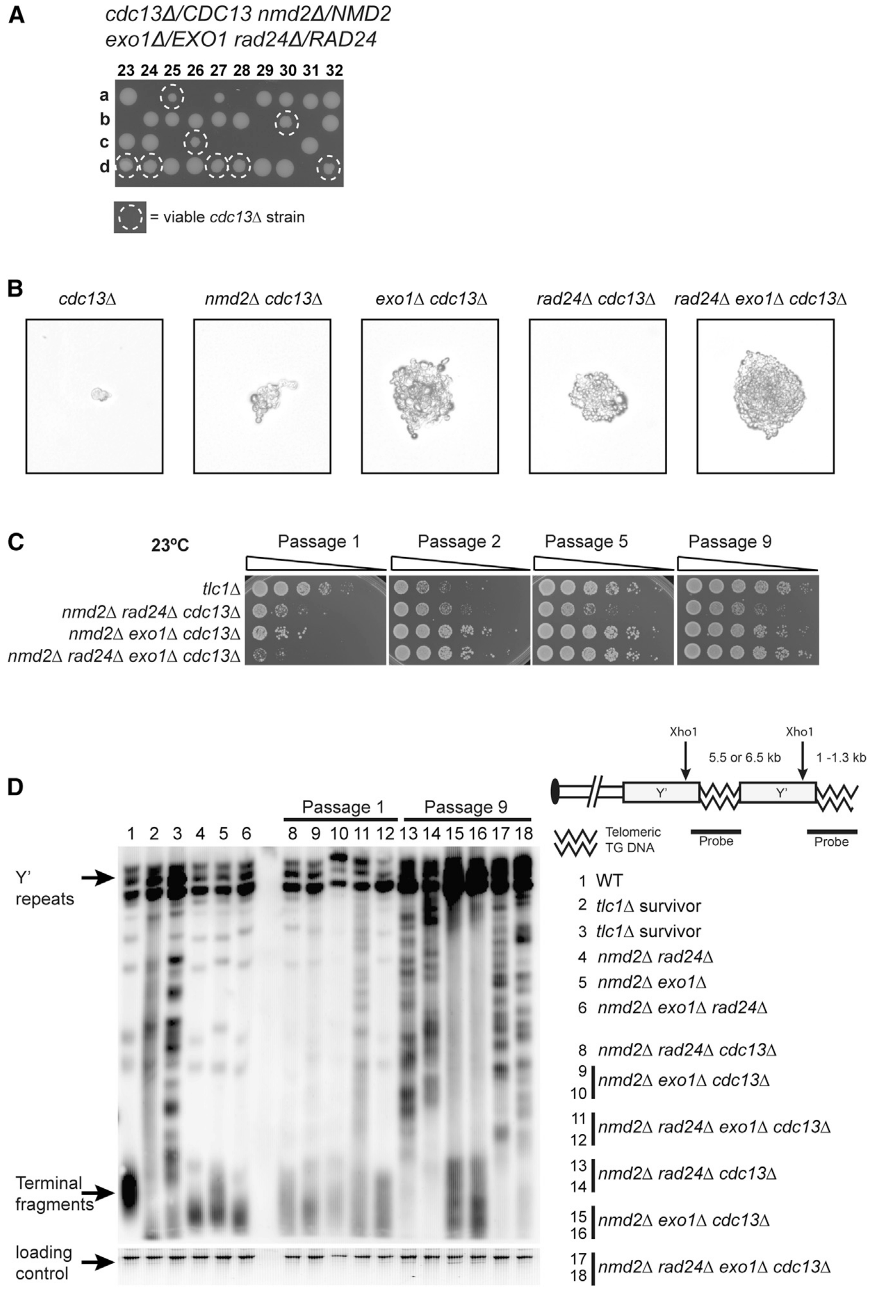

Figure 3. Bypass of *cdc13Δ*

(A) *NMD2/nmd2Δ EXO1/exo1Δ RAD24/rad24Δ CDC13/cdc13Δ* diploids were sporulated. Tetrads were dissected onto YEPD plates, and spores were allowed to form colonies for 5 days at 23°C before being photographed.

(B) Following germination of spores in (A), microcolonies were photographed using a 20× objective on a Microtec microscope and reproduced at the same scale. A representative subset of microcolonies is shown.

(C) Strains of the genotypes indicated were repeatedly passaged by toothpick every 4 days at 23°C. At the indicated times, 2 ml liquid cultures were grown overnight, serially diluted, spotted onto YEPD plates, and incubated for 2 days before being photographed.

(D) Genomic DNA was isolated from the yeast strains indicated, and telomere structures were analyzed by Southern blotting using a Y′ and TG probe. SYBR Safe was used as a loading control. See also Figures S2 and S3.

this, we deleted *CDC13* in a diploid strain that carried heterozygous deletions of *UPF1*, *EXO1*, and *RAD24*. We sporulated the diploid, dissected tetrads, and germinated the spores. Consistent with our hypothesis, 100% of *nmd2Δ rad24Δ cdc13Δ, nmd2Δ exo1Δ cdc13Δ*, and *nmd2Δ rad24Δ exo1Δ cdc13Δ* spores formed visible colonies, whereas all other *cdc13Δ* genotypes did not (Figures 3A and S2A). Inviable *cdc13Δ* strains formed microcolonies, and the sizes of microcolonies were increased by *nmd2Δ*, *exo1Δ*, or *rad24Δ* mutations (Figures 3B and S2B), just as the deletions improved fitness of *cdc13-1* cells at semipermissive temperatures (Figure 1B). Therefore, combining disruptions affecting NMD with those affecting *EXO1* and *RAD24* can permit cell division in the absence of Cdc13.

Because some *cdc13Δ* genotypes form visible colonies, whereas other *cdc13Δ* genotypes form only microscopic colonies, we wondered whether cells in large *cdc13Δ* colonies might eventually stop dividing. To examine fitness over time, we subcultured viable *cdc13Δ* strains for many passages and measured fitness by spot test. Fitness of the *cdc13Δ* strains increased rather than decreased with time (Figure 3C), similar to telomerase-deficient strains (*tlc1Δ*), which escape senescence and maintain telomere length by mechanisms independent of telomerase (Lundblad and Blackburn, 1993; Wellinger and Zakian, 2012). Consistent with this similarity, when we examined telomere structures of *cdc13Δ* strains they were altered by passage 9 and showed rearrangements like telomerase-deficient survivors (Figure 3D). We conclude that *cdc13Δ* cells are viable indefinitely and rearrange their telomere structures like telomerase-deficient cells.

Given that *cdc13Δ* cells rearranged telomeres like telomerase-deficient *tlc1Δ* cells, we wondered if they needed functional telomerase in order to divide, as *cdc13Δ pif1Δ exo1Δ* have been demonstrated to depend on telomerase for survival (Dewar and Lydall, 2010). We germinated spores derived after introducing a *tlc1Δ* disruption into a diploid strain containing heterozygous deletions of *CDC13*, *NMD2*, *RAD24*, or *EXO1*. We found viable *cdc13Δ tlc1Δ* strains when *nmd2Δ* and *exo1Δ*, *rad24Δ*, or *exo1Δ rad24Δ* were present (Figure S3A). Furthermore such

strains could be cultured for many passages, showed increased fitness over time and altered telomere structure like telomerase-deficient survivors (Figures S3B and S3C). We conclude that *nmd2Δ cdc13Δ* strains use telomerase-independent mechanisms to maintain telomere length.

The Requirement for *STN1 and TEN1* Cannot Be Bypassed

To test whether yeast cells survive without the Stn1 or Ten1, the other components of the CST complex, we introduced *stn1Δ* or *ten1Δ* disruptions into the diploid strain containing heterozygous deletions of *NMD2*, *RAD24*, or *EXO1*. In contrast to what was found with *cdc13Δ*, we could not identify any visible *stn1Δ* or *ten1Δ* colonies (Figures 4, S4A, and S4B). Interestingly, germinated *stn1Δ* and *ten1Δ* spores often formed microcolonies like some of the *cdc13Δ* genotypes (Figures 4 and 3B). Therefore, *stn1Δ* and *ten1Δ* cells sometimes undergo a limited number of cell divisions but cannot divide indefinitely, irrespective of the status of *NMD2*, *RAD24*, or *EXO1*. Similarly, *cdc13Δ*, *nmd2Δ cdc13Δ*, *exo1Δ cdc13Δ*, *rad24Δ cdc13Δ*, and *exo1Δ rad24Δ cdc13Δ* cells sometimes undergo a few cell divisions before stopping division (Figure 3B). We note that others have reported that *stn1Δ rad24Δ* microcolonies are smaller than *cdc13Δ rad24Δ* microcolonies, which is consistent with our data (Paschini et al., 2012). In summary, all these microcolony patterns suggest that *stn1Δ* and *ten1Δ* strains have similar but more severe growth defects than *cdc13Δ* strains.

One explanation for the fitness differences between *cdc13Δ* and *stn1Δ* or *ten1Δ* strains was that fitness differences were not due to CST defects per se but instead because important genes adjacent to *STN1* and *TEN1* were affected in deletion strains (Ben-Shitrit et al., 2012). However, this is not the case because the essential functions missing in *stn1Δ* and *ten1Δ* strains could be rescued by expressing the missing *STN1* or *TEN1* genes on plasmids (Figure S4C). Furthermore, the strains relying on plasmid-borne *STN1* or *TEN1* could not lose such plasmids (Figure S4D). Given that several defined genetic backgrounds allow growth of *cdc13Δ* but not of *stn1Δ* or *ten1Δ* strains, this strongly implies that Stn1 and Ten1 are more critical for cell viability than Cdc13.

Our experiments show that budding yeast cells defective in NMD and Exo1 or Rad24 can grow indefinitely without Cdc13 and telomerase, but that in such cells telomere function is compromised. However, cells with otherwise identical genetic backgrounds cannot grow in the absence of Stn1 or Ten1. The simplest explanation for these observations is that Stn1 and Ten1 play additional roles to Cdc13 in maintaining budding yeast cell viability. Consistent with this data, other experiments have shown that truncated and overexpressed versions of Stn1/Ten1 can bypass the need for Cdc13 (Petreaca et al., 2006, 2007; Gasparyan et al., 2009).

It has been shown that the *nmd2Δ* telomere phenotype is due, at least in part, to elevated Stn1 levels. Specifically, overexpression of *STN1*, or simultaneous overexpression of *STN1* and *TEN1*, leads to short telomeres of a similar length to *nmd2Δ* mutants (Dahlseid et al., 2003). Therefore, we wondered whether growth of *cdc13Δ* cells depended on Stn1 and/or Ten1 overproduction. To test this hypothesis, we examined a different genetic background that was not expected to affect Stn1 levels.

Pif1 is a helicase that is active at telomeres and deletion of *PIF1* and *EXO1* also permits deletion of *CDC13* (Dewar and Lydall, 2010). We repeated previous experiments and were able to generate viable strains from germinated *cdc13Δ pif1Δ exo1Δ* spores. However, we were unable to generate equivalent *stn1Δ* or *ten1Δ* strains (Figures 5A–5C and S5), reproducing what was found in other genetic backgrounds (Figure 4). These results strongly suggested that overexpression of Stn1 was not necessary to bypass Cdc13 function. However, it remained possible that *pif1Δ* or *exo1Δ* mutations caused increased Stn1 or Ten1 levels. Therefore, we measured Stn1 and Ten1 RNA expression levels in *pif1Δ*, *exo1Δ*, and *pif1Δ exo1Δ* strains, using quantitative RT-PCR (qRT-PCR). In these strains, levels of *STN1* and *TEN1* RNA were not significantly different from wild-type (Figures 5D and 5E), whereas, as expected, levels of *STN1* and *TEN1* RNAs were increased by an *nmd2Δ* mutation. Finally, it was possible that Stn1 or Ten1 might be transcriptionally induced by the response to telomere uncapping in *cdc13Δ* cells. However, this is not the case because there is no significant increase in *STN1* and *TEN1* RNA levels in *cdc13-1* strains grown at high temperatures (Greenall et al., 2008). We conclude that bypass of the requirement for Cdc13 does not depend on *nmd2Δ*-dependent overexpression of *STN1* and/or *TEN1*. Instead, our data suggest that at normal levels of expression Stn1 and Ten1 can, in some circumstances, function without Cdc13 to maintain viability of yeast cells.

Stn1, Ten1, and Cdc13 Can Bind Telomeric DNA at Different Ratios

Our experiments show that Stn1 and Ten1 contribute to yeast cell viability in conditions when Cdc13 is not required. To see if the essential function provided by Stn1 or Ten1 was at telomeres, we asked whether disabling the NMD pathway affected the ratio of CST components at telomeres. To investigate this, we used a chromatin immunoprecipitation (ChIP) assay to measure binding of myc-tagged *STN1*, *TEN1*, and *CDC13* to telomeric DNA, in wild-type or *nmd2Δ* backgrounds. We observed about a 10-fold increase in binding of Stn1 and a 5-fold increase for Ten1 to telomeres in *nmd2Δ* mutants but only a 2-fold increase in the levels of Cdc13 (Figures 6A–6C). We conclude that Cdc13, Stn1, and Ten1, the components of the CST complex, can bind telomeres at different ratios.

Given that we were able to delete Cdc13, but could not delete Stn1 or Ten1, the other two components of the CST complex, this suggests that Stn1 or Ten1 might help cap the telomere in the complete absence of Cdc13. We tested this hypothesis using a ChIP assay. We found that Stn1-Myc was indeed bound to telomeric DNA in a Cdc13-independent manner in an *nmd2Δ exo1Δ rad24Δ cdc13Δ* strain (Figure 6D). The level of Stn1 binding to telomeres was lower in the *cdc13Δ* strain compared to the *CDC13*$^{+}$ strain; this could be due to the *cdc13Δ* cells having dramatically rearranged telomeres. We did not find evidence of Ten1 binding to telomeres in the absence of Cdc13 (Figure 6E). However, Ten1 enrichment at telomeres was also relatively weak in the *nmd2Δ exo1Δ rad24Δ* strain, and it may be that any binding is below our detection limit. RPA is another ssDNA

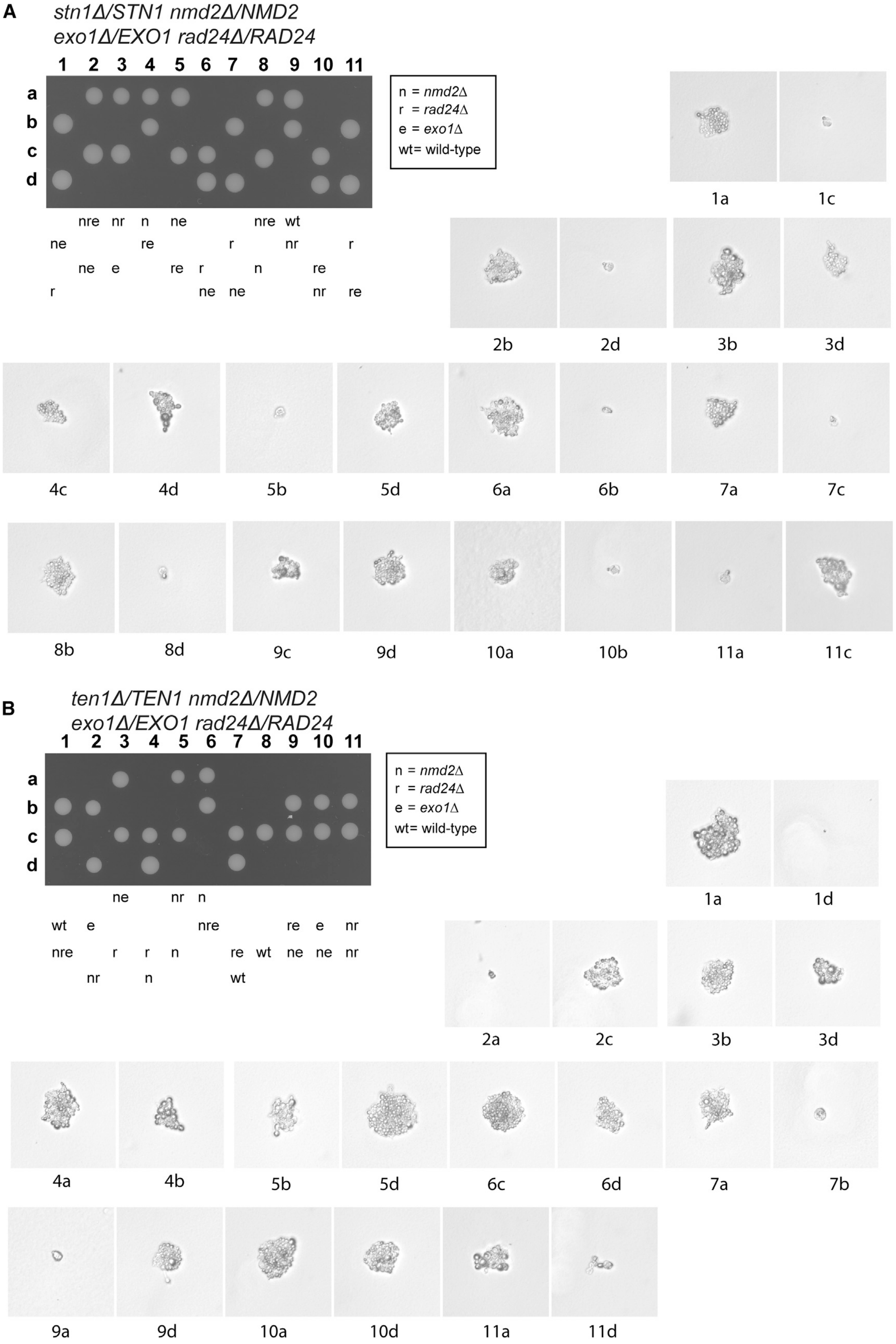

(legend on next page)

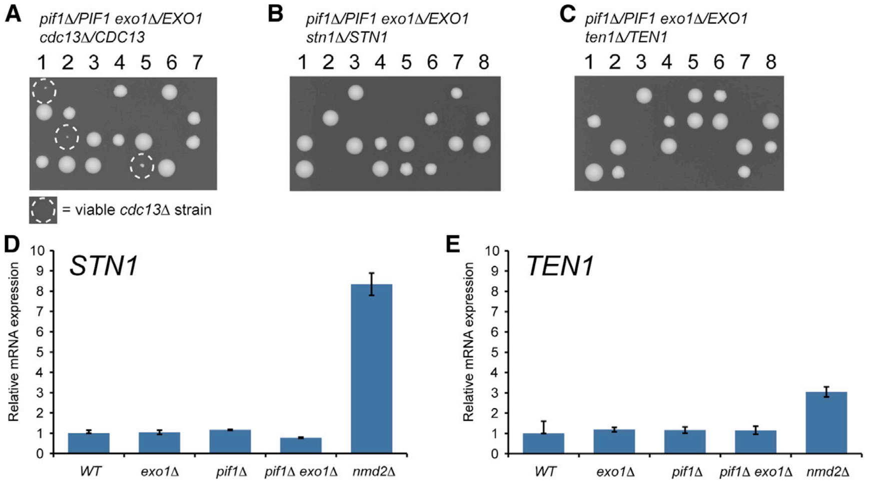

Figure 5. Bypass of Cdc13 in a *pif1Δ exo1Δ* Strain Does Not Depend on Overexpression of Stn1 and Ten1

(A–C) *PIF1/pif1Δ EXO1/exo1Δ CDC13/cdc13Δ*, *PIF1/pif1Δ EXO1/exo1Δ STN1/stn1Δ*, and *PIF1/pif1Δ EXO1/exo1Δ TEN1/ten1Δ* diploids were sporulated. Tetrads were dissected onto YEPD plates, and spores were allowed to form colonies for 5 days at 23°C before being photographed. See also Figure S5.

(D and E) qRT-PCR analysis of Stn1 and Ten1 RNA expression levels in the strains indicated. A single wild-type (WT) strain was given the value of 1, and the error bar indicates the value of the other wild-type strain. All other genotypes are expressed relative to the single wild-type strain, the mean of two independent strains is shown, and error bars indicate the individual value of each strain.

See also Figure S5.

binding protein, binds at telomeres, and is therefore likely to compete with Cdc13 as a telomeric ssDNA binding protein. Consistent with this hypothesis, we measured more RPA bound to telomeres in the absence of Cdc13 (Figure 6F). This suggests that in the absence of Cdc13, RPA can bind telomeric DNA and that RPA cooperates with Stn1, Ten1, and other proteins to cap the telomere. We conclude that Stn1 can bind telomeres in the absence of Cdc13.

DISCUSSION

We have shown that NMD acts in a parallel pathway to the Exo1 and Rad24 DDR proteins to inhibit growth of yeast cells with defective telomeres. Furthermore, we show that NMD, like Exo1 and Rad24, affects the level of telomeric ssDNA. Remarkably, we find that the requirement for *CDC13* can be robustly bypassed in 100% of cells with *nmd2Δ* and *exo1Δ* or *rad24Δ* mutations. Viable *cdc13Δ* strains can be cultured for many passages, and the telomeres in such cells resemble those of telomerase-deficient survivors and still bind Stn1. In contrast, none of the four genetic backgrounds that allow robust bypass of *cdc13Δ* allowed bypass of *stn1Δ* or *ten1Δ*.

Cdc13, along with Stn1 and Ten1, has been proposed to form an essential heterotrimeric telomeric ssDNA binding complex analogous to RPA, the ssDNA binding complex (Gao et al., 2007). The CST/RPA model is attractive for many reasons, perhaps most notably because all three CST subunits are, like the RPA subunits, essential for yeast cell viability, and all three contribute to telomere protection. However, we have identified several defined genetic backgrounds that permit deletion of *CDC13*, but none of these permit deletion of *STN1* or *TEN1*. The simplest explanation for these data is that Stn1 and Ten1 play Cdc13-independent roles at the telomere, or elsewhere. We show that *STN1* and *TEN1* binding to telomeric DNA increases more than Cdc13 in *nmd2Δ* strains, which suggests that Stn1 and Ten1 can bind telomeric DNA without Cdc13. Indeed, we also show that Stn1 binds to telomeric DNA in the absence of Cdc13. Consistent with our data, others have shown that C-terminal truncations of Stn1, which disrupt the Stn1-Cdc13 interaction, are sufficient to support cell viability and telomere function (Petreaca et al., 2007). Interestingly, Stn1 overproduction inactivates the S phase checkpoint in budding yeast, and, although the biochemical mechanism explaining this interaction is not known, it is tempting to speculate that some aspect of this checkpoint inhibition function is critical for Stn1 function (Gasparyan et al., 2009). We conclude that budding yeast Cdc13—the largest component of the CST complex—contributes to a subset of the essential functions performed by its smaller partners, Stn1 and Ten1.

Ten1 was the last of the budding yeast CST components to be identified, in 2001 (Grandin et al., 2001). It was only much more recently that orthologs of CST components were identified in higher eukaryotes (Giraud-Panis et al., 2010). Our data from budding yeast, showing that *STN1* and *TEN1* are critical for cell viability in conditions when *CDC13* is not, are consistent with data from other organisms, suggesting that this pattern might be universally the case in eukaryotes. For example, so far, no ortholog of Cdc13 has yet been reported in fission yeast but orthologs of both Stn1 and Ten1 have been identified (Jain and Cooper, 2010). Also, mutations in human *CTC1*, the ortholog of *CDC13*, are found in a number of diseases associated with telomere defects (Coats plus, *dyskeratosis congenita* and CRMCC); however, no equivalent mutations in *STN1* or *TEN1* have yet been identified in the same cohorts of patients (Anderson et al., 2012; Polvi et al., 2012; Walne et al., 2012). Perhaps mutations in Stn1 or

Figure 4. *stn1Δ* and *ten1Δ* Mutants Form Microcolonies

(A and B) *NMD2/nmd2Δ EXO1/exo1Δ RAD24/rad24Δ STN1/stn1Δ* and *NMD2/nmd2Δ EXO1/exo1Δ RAD24/rad24Δ TEN1/ten1Δ* diploids were sporulated. Tetrads were dissected onto YEPD plates, and spores were allowed to form colonies for 5 days at 23°C before being photographed (see also Figure S4). Following germination of spores, microcolonies were photographed using a 20× objective on a Microtec microscope and reproduced at the same scale.

See also Figure S4. We are uncertain about the genotypes of individual microcolonies as we cannot establish which gene deletions were inherited by each spore (in contrast to Figure 3B).

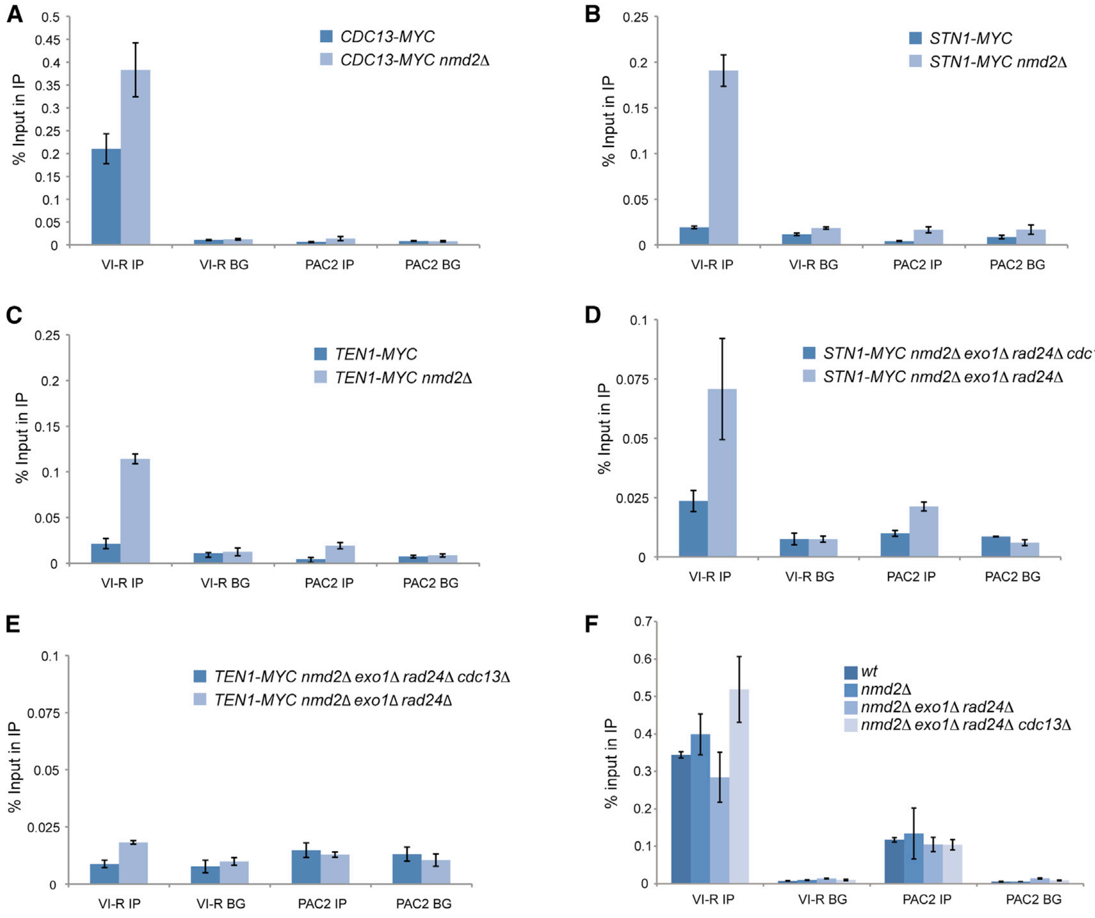

Figure 6. Altered Stoichiometry of CST Components at Telomeres

(A–E) ChIP analysis of Cdc13-13Myc, Stn1-13Myc, and Ten1-13Myc binding to the VI-R telomere and the internal locus *PAC2* on chromosome V. Cultures of each genotype were grown at 23°C, and cells were harvested in exponential phase. Duplicate samples were immunoprecipitated with a Myc antibody (IP) or a nonspecific IgG control (BG). ChIP samples were measured in triplicate by qPCR, and group means are shown with error bars indicating SD.

(F) ChIP analysis of RPA binding to the VI-R telomere and the internal locus *PAC2* on chromosome V. ChIP was conducted as in (A)–(E) using an anti-*S. cerevisiae* RPA antibody (IP) or a nonspecific IgG control (BG). ChIP samples were measured in triplicate by qPCR, and group means are shown with error bars indicating SD.

Ten1 in humans cause stronger phenotypes that are not tolerated.

We have previously shown that some *cdc13Δ* strains can also be deleted of *STN1* (Zubko and Lydall, 2006). *stn1Δ* strains grew less well than the parental (*cdc13Δ*) strains, and we were unable to identify any *ten1Δ* strains. These data, and those we report here, show that a functional telomere is very flexible in terms of the proteins it contains. The possibility remains that conditions will be identified that permit bypass of Stn1 and or Ten1 but not of Cdc13. A better understanding of the functions of Cdc13, Stn1, and Ten1 at telomeres will be important to see if this is likely. As it stands, our data suggest there is a functional hierarchy of CST subunit function in budding yeast with Ten1 more critical than Stn1, which is more critical than Cdc13.

If Stn1 and Ten1 function at eukaryotic telomeres in the absence of Cdc13, then how do they do so? Because Stn1 and Ten1 have low affinity for telomeric DNA (in comparison with Cdc13), one simple explanation is that Stn1 and Ten1 bind and cap the telomere via interactions with any of the numerous other telomere binding proteins or RNAs. The idea that Stn1 interacts with proteins other than Cdc13 to perform essential functions is consistent with data showing that the Ten1 interaction domain of Stn1 is much more critical for cell viability than the Cdc13 interaction domain (Petreaca et al., 2007). Stn1/Ten1 might interact with one or more than one of numerous other proteins found at budding yeast telomeres, and elsewhere, including Rap1, Rif1, Rif2, Ku, MRX, Tel1, Telomerase, Sir proteins, RPA, and DNA polymerase alpha. We tested a model in which subunits of RPA formed heterotrimers with CST subunits, but we could obtain no strong evidence for such a model (data not shown). However, we did observe increased binding of RPA to telomeres in the absence of Cdc13. Both budding yeast and mammalian CST components interact with Pol α primase and in yeast this interaction has been shown to promote telomere capping (Grossi et al., 2004; Gasparyan et al., 2009; Qi and Zakian, 2000; Anderson et al.,

2012). In mammalian cells, CST components facilitate the replication of telomeric lagging-strand DNA (Sun et al., 2011; Nakaoka et al., 2012). It will be interesting to determine how telomeres are capped and replication is completed in the absence of Cdc13.

Finally, given that CST and NMD play important roles in telomeres in yeast and humans, the genetic interactions we report in yeast may identify useful avenues to pursue for developing future treatments for the human diseases in which *CTC1* is affected (Gu and Chang, 2013). Premature termination codons are responsible for around 10% of inherited human diseases and pharmaceuticals targeting NMD have been identified. If we extrapolate from the yeast experiments to human cells, it is conceivable that reducing NMD function pharmaceutically might compensate for loss of CTC1 function in patients.

EXPERIMENTAL PROCEDURES

Yeast Strains

All strains are in the W303 background and are *RAD5*$^{+}$ (Supplemental Experimental Procedures, list 1). Gene disruptions of *CDC13*, *STN1*, and *TEN1* were created by inserting a hygromycin cassette into a diploid using one step PCR, primers indicated in Supplemental Experimental Procedures (list 2) and a pAG32 plasmid harboring HPHMX4 (Goldstein and McCusker, 1999) (Supplemental Experimental Procedures, list 3). Gene disruptions were confirmed by PCR. *STN1* and *TEN1* rescue plasmids were created by PCR-based gap repair of plasmid pDL1466 (see Supplemental Experimental Procedures, list 3, for plasmid details).

Yeast Growth Assays

Single colonies were inoculated into 2 ml of YEPD+adenine and grown in tubes at 23°C overnight until saturation. Six-fold serial dilution series of the cultures were spotted onto plates using a 48-prong replica-plating device. Plates were incubated for 2–3 days at temperatures indicated before being photographed. For cycling temperature assays plates were incubated at 23°C for 4 hr then 36°C for 4 hr, and this was repeated three times before colonies were allowed to form at 23°C. For passage experiments, several colonies were pooled with a toothpick and restruck onto YEPD plates.

ChIP

Chromatin immunoprecipitation was performed essentially as previously described (Dubarry et al., 2011); cells were additionally crosslinked with 2 mM EGS. Mouse anti-myc 9E10 (ab32, Abcam) or anti-*Saccharomyces cerevisiae* RPA (AS07 214, Agrisera) or rabbit anti-goat immunoglobulin (Ig) G (ab97096, Abcam) antibodies were used for immunoprecipitations. Immunoprecipitated DNA was isolated using 10% Chelex (Bio-Rad) and quantified by qPCR using the SYBR Green pPCR SuperMIX-UDG w/ROX kit (Invitrogen, 11744500). Primers used for PCR are described in the Supplemental Experimental Procedures, list 2.

Synchronous Cultures and QAOS

Synchronous culture experiments and viability assay were carried out in strains containing *bar1Δ cdc15-2* mutations and were performed as described (Zubko et al., 2006). Quantitative amplification of ssDNA was carried out as described (Holstein and Lydall, 2012).

In-Gel Assay

In-gel assays were performed as previously described (Dewar and Lydall, 2012). The Cy5-labeled oligonucleotide (M2188) was detected on a GE Healthcare Typhoon Trio imager. The agarose gel was poststained using SYBR Safe, and total DNA was detected using a FUJI LAS-4000 imager. ssDNA was quantified using ImageJ and normalized relative to the loading control. The final fold change is relative to the 0 hr time point of each strain.

Microcolonies

After germination for 5 days at 23°C, colonies were photographed using a 20× objective on a Microtec microscope. An image was taken of each microcolony, and images are reproduced at the same scale for direct comparison.

Analysis of Telomere Structure

Southern blot analysis was performed essentially as previously described (Maringele and Lydall, 2004). Genomic DNA was cut with XhoI (New England Biolabs), run overnight on a 0.8% agarose gel, and transferred to a positively charged nylon membrane. The membrane was hybridized with a 1 kbp Y′ and TG probe, obtained by digesting pDL987 with XhoI and BamHI. The probe was labeled, and the blot was hybridized and immunologically detected using the DIG-High Prime Labeling and Detection Kit (Roche, 11585614910). The probe was visualized using a FUJI LAS-4000 imager.

Quantitative RT-PCR

RNA isolation was performed essentially as described (Collart and Oliviero, 2001). RNA was further purified using the RNEasy Mini Kit (QIAGEN, 74104) and by DNase I digestion (Invitrogen, 18068-015). Quantitative RT-PCR was carried out using the Superscript III Platinum SYBR Green One-Step qRT-PCR kit (Invitrogen, 11736-059). RNA samples were normalized relative to the *BUD6* loading control.

SUPPLEMENTAL INFORMATION

Supplemental Information includes Supplemental Experimental Procedures and five figures and can be found with this article online at http://dx.doi.org/10.1016/j.celrep.2014.04.017.

AUTHOR CONTRIBUTIONS

E.-M.H. initiated this project and generated the data in Figures 1, 2, and 4. K.R.M.C. continued with the project and contributed, with Eva Holstein, to Figures 3 and 5. K.R.M.C. generated the data in Figure 6. All authors contributed to experimental design and writing the paper.

ACKNOWLEDGMENTS

We thank all members of the lab for input, in particular, M. Dubarry for the plasmid pDL1466, C. Lawless and J. Heydari for statistical advice, and J. Rodrigues for help with quantification. We thank B. Lightowlers and C. Nugent for comments on the manuscript. The work was supported by the Wellcome Trust (WT093088MA) and the BBSRC (BB/F006039/1 and BBF016980/1).

Received: March 6, 2013
Revised: March 20, 2014
Accepted: April 10, 2014
Published: May 15, 2014

REFERENCES

Addinall, S.G., Holstein, E.M., Lawless, C., Yu, M., Chapman, K., Banks, A.P., Ngo, H.P., Maringele, L., Taschuk, M., Young, A., et al. (2011). Quantitative fitness analysis shows that NMD proteins and many other protein complexes suppress or enhance distinct telomere cap defects. PLoS Genet. *7*, e1001362.

Anderson, B.H., Kasher, P.R., Mayer, J., Szynkiewicz, M., Jenkinson, E.M., Bhaskar, S.S., Urquhart, J.E., Daly, S.B., Dickerson, J.E., O'Sullivan, J., et al. (2012). Mutations in CTC1, encoding conserved telomere maintenance component 1, cause Coats plus. Nat. Genet. *44*, 338–342.

Azzalin, C.M., Reichenbach, P., Khoriauli, L., Giulotto, E., and Lingner, J. (2007). Telomeric repeat containing RNA and RNA surveillance factors at mammalian chromosome ends. Science *318*, 798–801.

Ben-Shitrit, T., Yosef, N., Shemesh, K., Sharan, R., Ruppin, E., and Kupiec, M. (2012). Systematic identification of gene annotation errors in the widely used yeast mutation collections. Nat. Methods *9*, 373–378.

Bidou, L., Allamand, V., Rousset, J.P., and Namy, O. (2012). Sense from nonsense: therapies for premature stop codon diseases. Trends Mol. Med. *18*, 679–688.

Bunting, S.F., Callén, E., Wong, N., Chen, H.T., Polato, F., Gunn, A., Bothmer, A., Feldhahn, N., Fernandez-Capetillo, O., Cao, L., et al. (2010). 53BP1 inhibits homologous recombination in Brca1-deficient cells by blocking resection of DNA breaks. Cell *141*, 243–254.

Casteel, D.E., Zhuang, S., Zeng, Y., Perrino, F.W., Boss, G.R., Goulian, M., and Pilz, R.B. (2009). A DNA polymerase-αmiddle dotprimase cofactor with homology to replication protein A-32 regulates DNA replication in mammalian cells. J. Biol. Chem. *284*, 5807–5818.

Chandra, A., Hughes, T.R., Nugent, C.I., and Lundblad, V. (2001). Cdc13 both positively and negatively regulates telomere replication. Genes Dev. *15*, 404–414.

Chen, L.Y., Redon, S., and Lingner, J. (2012). The human CST complex is a terminator of telomerase activity. Nature *488*, 540–544.

Chen, L.Y., Majerská, J., and Lingner, J. (2013). Molecular basis of telomere syndrome caused by CTC1 mutations. Genes Dev. *27*, 2099–2108.

Collart, M.A., and Oliviero, S. (2001). Preparation of yeast RNA. Curr. Protoc. Mol. Biol., Published online May 1, 2001 http://dx.doi.org/10.1002/0471142727.mb1312s23.

Dahlseid, J.N., Lew-Smith, J., Lelivelt, M.J., Enomoto, S., Ford, A., Desruisseaux, M., McClellan, M., Lue, N., Culbertson, M.R., and Berman, J. (2003). mRNAs encoding telomerase components and regulators are controlled by UPF genes in Saccharomyces cerevisiae. Eukaryot. Cell *2*, 134–142.

Dewar, J.M., and Lydall, D. (2010). Pif1- and Exo1-dependent nucleases coordinate checkpoint activation following telomere uncapping. EMBO J. *29*, 4020–4034.

Dewar, J.M., and Lydall, D. (2012). Simple, non-radioactive measurement of single-stranded DNA at telomeric, sub-telomeric, and genomic loci in budding yeast. Methods Mol. Biol. *920*, 341–348.

Dubarry, M., Loïodice, I., Chen, C.L., Thermes, C., and Taddei, A. (2011). Tight protein-DNA interactions favor gene silencing. Genes Dev. *25*, 1365–1370.

Gao, H., Cervantes, R.B., Mandell, E.K., Otero, J.H., and Lundblad, V. (2007). RPA-like proteins mediate yeast telomere function. Nat. Struct. Mol. Biol. *14*, 208–214.

Garvik, B., Carson, M., and Hartwell, L. (1995). Single-stranded DNA arising at telomeres in cdc13 mutants may constitute a specific signal for the RAD9 checkpoint. Mol. Cell. Biol. *15*, 6128–6138.

Gasparyan, H.J., Xu, L., Petreaca, R.C., Rex, A.E., Small, V.Y., Bhogal, N.S., Julius, J.A., Warsi, T.H., Bachant, J., Aparicio, O.M., and Nugent, C.I. (2009). Yeast telomere capping protein Stn1 overrides DNA replication control through the S phase checkpoint. Proc. Natl. Acad. Sci. USA *106*, 2206–2211.

Giraud-Panis, M.J., Teixeira, M.T., Géli, V., and Gilson, E. (2010). CST meets shelterin to keep telomeres in check. Mol. Cell *39*, 665–676.

Goldstein, A.L., and McCusker, J.H. (1999). Three new dominant drug resistance cassettes for gene disruption in Saccharomyces cerevisiae. Yeast *15*, 1541–1553.

Grandin, N., Damon, C., and Charbonneau, M. (2001). Ten1 functions in telomere end protection and length regulation in association with Stn1 and Cdc13. EMBO J. *20*, 1173–1183.

Greenall, A., Lei, G., Swan, D.C., James, K., Wang, L., Peters, H., Wipat, A., Wilkinson, D.J., and Lydall, D. (2008). A genome wide analysis of the response to uncapped telomeres in budding yeast reveals a novel role for the NAD+ biosynthetic gene BNA2 in chromosome end protection. Genome Biol. *9*, R146.

Grossi, S., Puglisi, A., Dmitriev, P.V., Lopes, M., and Shore, D. (2004). Pol12, the B subunit of DNA polymerase alpha, functions in both telomere capping and length regulation. Genes Dev. *18*, 992–1006.

Gu, P., and Chang, S. (2013). Functional characterization of human CTC1 mutations reveals novel mechanisms responsible for the pathogenesis of the telomere disease Coats plus. Aging Cell *12*, 1100–1109.

Guan, Q., Zheng, W., Tang, S., Liu, X., Zinkel, R.A., Tsui, K.W., Yandell, B.S., and Culbertson, M.R. (2006). Impact of nonsense-mediated mRNA decay on the global expression profile of budding yeast. PLoS Genet. *2*, e203.

Holstein, E.M., and Lydall, D. (2012). Quantitative amplification of single-stranded DNA. Methods Mol. Biol. *920*, 323–339.

Isken, O., and Maquat, L.E. (2008). The multiple lives of NMD factors: balancing roles in gene and genome regulation. Nat. Rev. Genet. *9*, 699–712.

Jain, D., and Cooper, J.P. (2010). Telomeric strategies: means to an end. Annu. Rev. Genet. *44*, 243–269.

Lazzaro, F., Sapountzi, V., Granata, M., Pellicioli, A., Vaze, M., Haber, J.E., Plevani, P., Lydall, D., and Muzi-Falconi, M. (2008). Histone methyltransferase Dot1 and Rad9 inhibit single-stranded DNA accumulation at DSBs and uncapped telomeres. EMBO J. *27*, 1502–1512.

Lew, J.E., Enomoto, S., and Berman, J. (1998). Telomere length regulation and telomeric chromatin require the nonsense-mediated mRNA decay pathway. Mol. Cell. Biol. *18*, 6121–6130.

Lewis, K.A., Pfaff, D.A., Earley, J.N., Altschuler, S.E., and Wuttke, D.S. (2014). The tenacious recognition of yeast telomere sequence by Cdc13 is fully exerted by a single OB-fold domain. Nucleic Acids Res. *42*, 475–484.

Lue, N.F., Zhou, R., Chico, L., Mao, N., Steinberg-Neifach, O., and Ha, T. (2013). The telomere capping complex CST has an unusual stoichiometry, makes multipartite interaction with G-Tails, and unfolds higher-order G-tail structures. PLoS Genet. *9*, e1003145.

Lundblad, V., and Blackburn, E.H. (1993). An alternative pathway for yeast telomere maintenance rescues est1- senescence. Cell *73*, 347–360.

Maringele, L., and Lydall, D. (2004). EXO1 plays a role in generating type I and type II survivors in budding yeast. Genetics *166*, 1641–1649.

Mitton-Fry, R.M., Anderson, E.M., Theobald, D.L., Glustrom, L.W., and Wuttke, D.S. (2004). Structural basis for telomeric single-stranded DNA recognition by yeast Cdc13. J. Mol. Biol. *338*, 241–255.

Miyake, Y., Nakamura, M., Nabetani, A., Shimamura, S., Tamura, M., Yonehara, S., Saito, M., and Ishikawa, F. (2009). RPA-like mammalian Ctc1-Stn1-Ten1 complex binds to single-stranded DNA and protects telomeres independently of the Pot1 pathway. Mol. Cell *36*, 193–206.

Nakaoka, H., Nishiyama, A., Saito, M., and Ishikawa, F. (2012). Xenopus laevis Ctc1-Stn1-Ten1 (xCST) protein complex is involved in priming DNA synthesis on single-stranded DNA template in Xenopus egg extract. J. Biol. Chem. *287*, 619–627.

Nugent, C.I., Hughes, T.R., Lue, N.F., and Lundblad, V. (1996). Cdc13p: a single-strand telomeric DNA-binding protein with a dual role in yeast telomere maintenance. Science *274*, 249–252.

Paschini, M., Toro, T.B., Lubin, J.W., Braunstein-Ballew, B., Morris, D.K., and Lundblad, V. (2012). A naturally thermolabile activity compromises genetic analysis of telomere function in Saccharomyces cerevisiae. Genetics *191*, 79–93.

Petreaca, R.C., Chiu, H.C., Eckelhoefer, H.A., Chuang, C., Xu, L., and Nugent, C.I. (2006). Chromosome end protection plasticity revealed by Stn1p and Ten1p bypass of Cdc13p. Nat. Cell Biol. *8*, 748–755.

Petreaca, R.C., Chiu, H.C., and Nugent, C.I. (2007). The role of Stn1p in Saccharomyces cerevisiae telomere capping can be separated from its interaction with Cdc13p. Genetics *177*, 1459–1474.

Polvi, A., Linnankivi, T., Kivelä, T., Herva, R., Keating, J.P., Mäkitie, O., Pareyson, D., Vainionpää, L., Lahtinen, J., Hovatta, I., et al. (2012). Mutations in CTC1, encoding the CTS telomere maintenance complex component 1, cause cerebroretinal microangiopathy with calcifications and cysts. Am. J. Hum. Genet. *90*, 540–549.

Puglisi, A., Bianchi, A., Lemmens, L., Damay, P., and Shore, D. (2008). Distinct roles for yeast Stn1 in telomere capping and telomerase inhibition. EMBO J. *27*, 2328–2339.

Qi, H., and Zakian, V.A. (2000). The Saccharomyces telomere-binding protein Cdc13p interacts with both the catalytic subunit of DNA polymerase alpha and the telomerase-associated est1 protein. Genes Dev. *14*, 1777–1788.

Qian, W., Wang, J., Jin, N.N., Fu, X.H., Lin, Y.C., Lin, J.J., and Zhou, J.Q. (2009). Ten1p promotes the telomeric DNA-binding activity of Cdc13p: implication for its function in telomere length regulation. Cell Res. *19*, 849–863.

Qian, W., Fu, X.H., and Zhou, J.Q. (2010). Purification and characterization of Stn1p, a single-stranded telomeric DNA binding protein. Protein Expr. Purif. *73*, 107–112.

Sun, J., Yu, E.Y., Yang, Y., Confer, L.A., Sun, S.H., Wan, K., Lue, N.F., and Lei, M. (2009). Stn1-Ten1 is an Rpa2-Rpa3-like complex at telomeres. Genes Dev. *23*, 2900–2914.

Sun, J., Yang, Y., Wan, K., Mao, N., Yu, T.Y., Lin, Y.C., DeZwaan, D.C., Freeman, B.C., Lin, J.J., Lue, N.F., and Lei, M. (2011). Structural bases of dimerization of yeast telomere protein Cdc13 and its interaction with the catalytic subunit of DNA polymerase α. Cell Res. *21*, 258–274.

Surovtseva, Y.V., Churikov, D., Boltz, K.A., Song, X., Lamb, J.C., Warrington, R., Leehy, K., Heacock, M., Price, C.M., and Shippen, D.E. (2009). Conserved telomere maintenance component 1 interacts with STN1 and maintains chromosome ends in higher eukaryotes. Mol. Cell *36*, 207–218.

Walne, A., Bhagat, T., Kirwan, M., Gitaux, C., Desguerre, I., Leonard, N., Nogales, E., Vulliamy, T., and Dokal, I. (2012). Mutations in the telomere capping complex in bone marrow failure and related syndromes. Haematologica *97*, 524–528.

Wellinger, R.J., and Zakian, V.A. (2012). Everything you ever wanted to know about Saccharomyces cerevisiae telomeres: beginning to end. Genetics *191*, 1073–1105.

Zubko, M.K., and Lydall, D. (2006). Linear chromosome maintenance in the absence of essential telomere-capping proteins. Nat. Cell Biol. *8*, 734–740.

Zubko, M.K., Guillard, S., and Lydall, D. (2004). Exo1 and Rad24 differentially regulate generation of ssDNA at telomeres of Saccharomyces cerevisiae cdc13-1 mutants. Genetics *168*, 103–115.

Zubko, M.K., Maringele, L., Foster, S.S., and Lydall, D. (2006). Detecting repair intermediates in vivo: effects of DNA damage response genes on single-stranded DNA accumulation at uncapped telomeres in budding yeast. Methods Enzymol. *409*, 285–300.

Cell Reports, Volume *7*

Supplemental Information

Interplay between Nonsense-Mediated mRNA Decay and DNA Damage Response Pathways Reveals that Stn1 and Ten1 Are the Key CST Telomere-Cap Components

Eva-Maria Holstein, Kate R.M. Clark, and David Lydall

Figure S1

A

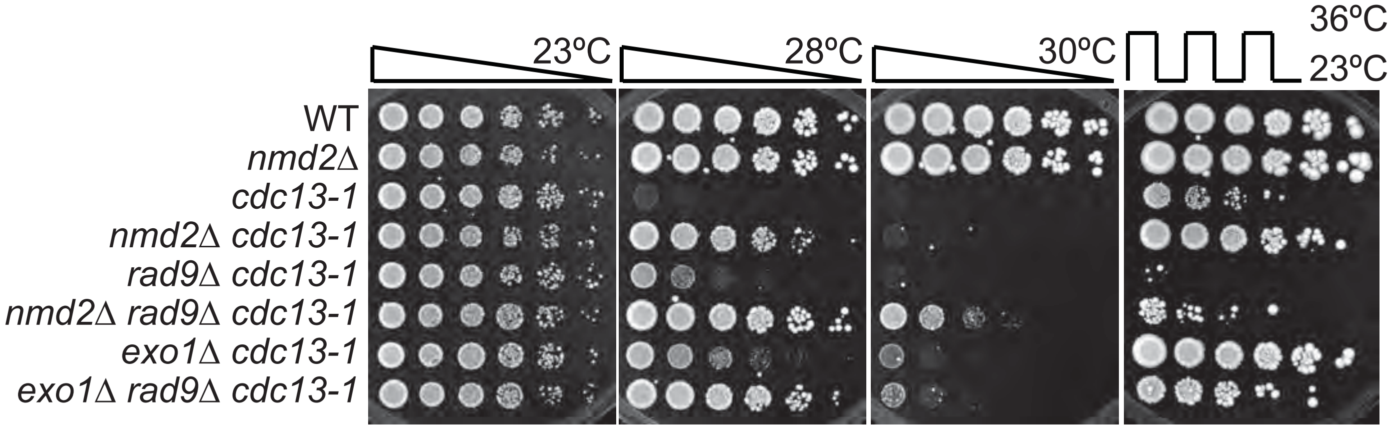

23ºC
28ºC
30ºC
36ºC
23ºC
WT
nmd2Δ
cdc13-1
nmd2Δ cdc13-1
rad9Δ cdc13-1
nmd2Δ rad9Δ cdc13-1
exo1Δ cdc13-1
exo1Δ rad9Δ cdc13-1

B

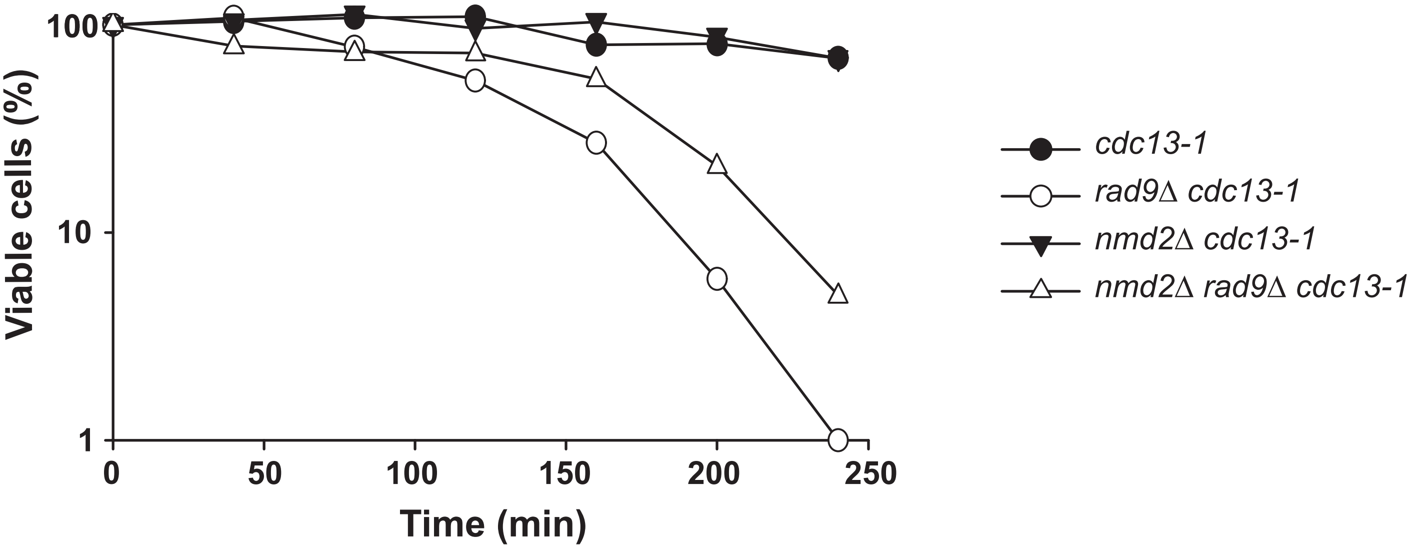

Viable cells (%)
100
10
1
0
50
100
150
200
250
Time (min)
cdc13-1
rad9Δ cdc13-1
nmd2Δ cdc13-1
nmd2Δ rad9Δ cdc13-1

Figure S1. Deletion of *NMD2* increases viability of *rad9*Δ *cdc13-1* mutants. Related to Figure 2.

(A) Saturated cultures were serially diluted across agar plates containing synthetic medium and were either grown at the temperature indicated for 3 days or cycled 3 times from 23°C for 4 hours to 36°C for 4 hours and then allowed to form colonies at 23°C for 3 days before being photographed. (B) Exponentially dividing cells containing *bar1*Δ and *cdc15-2* mutation were arrested at 23°C in G1 using alpha factor. Cultures were released from alpha factor and shifted to 36°C to induce telomere uncapping. Samples were taken at indicated time points, diluted, plated onto YEPD in duplicate and grown at 23°C for 3 days. Colony forming units were counted and viability was calculated using the dilution factor.

Figure S2

A *cdc13Δ/CDC13 nmd2Δ/NMD2 exo1Δ/EXO1 rad24Δ/RAD24*

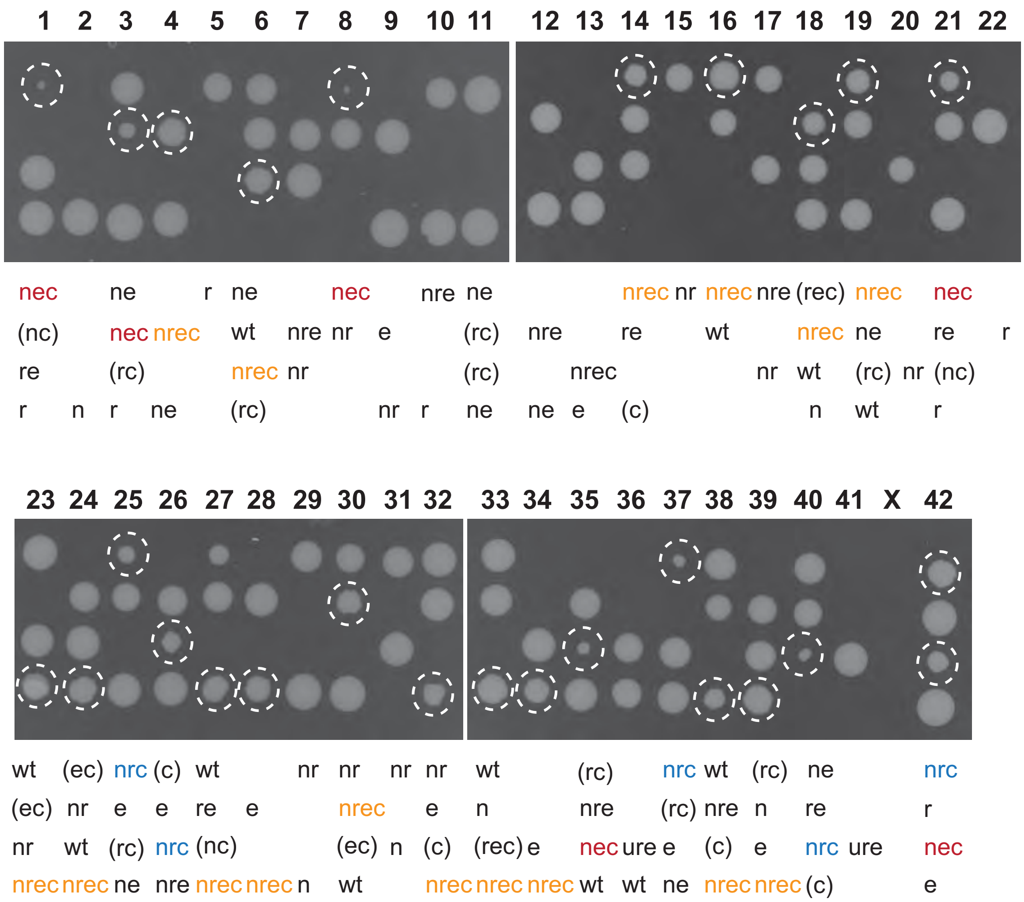

= viable *cdc13Δ* strain

n = *nmd2Δ*
r = *rad24Δ*
e = *exo1Δ*
c = *cdc13Δ*
() = inferred genotype
X = no spores dissected or spores not separated or spores lost

Genotype	Viability
cdc13Δ	0/4
nmd2Δ cdc13Δ	0/3
rad24Δ cdc13Δ	0/9
exo1Δ cdc13Δ	0/3
rad24Δ exo1Δ cdc13Δ	0/2
nmd2Δ rad24Δ cdc13Δ	5/5
nmd2Δ exo1Δ cdc13Δ	6/6
nmd2Δ rad24Δ exo1Δ cdc13	16/16

B

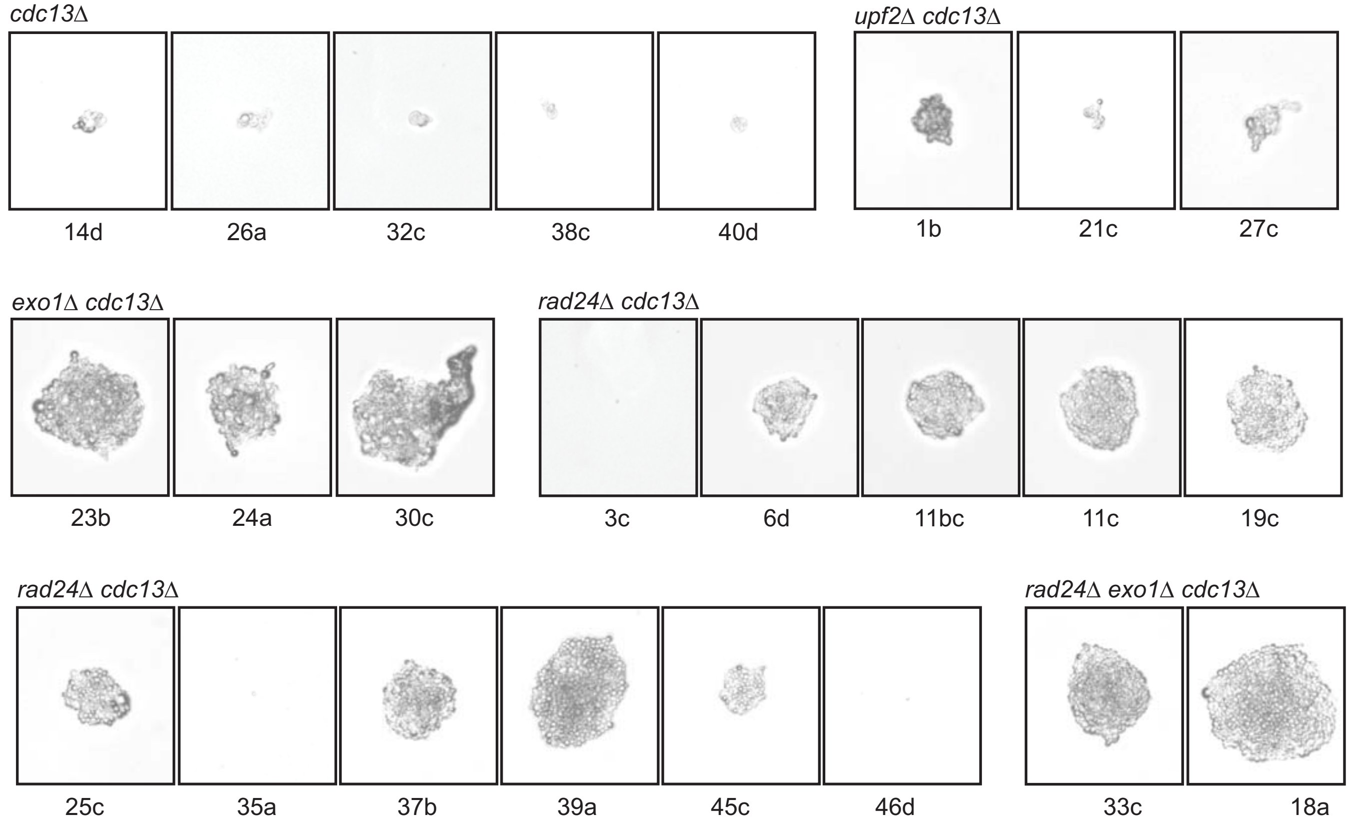

Figure S2. Growth of *cdc13*Δ strains. Related to Figure 3.

(A) Diploids containing heterozygous deletion mutations of *EXO1, NMD2, RAD24* and *CDC13* were sporulated. Tetrads were dissected and germinated on YEPD plates. Spores were allowed to form colonies for 5 days at 23°C before being photographed. Genotypes were determined by growth on appropriate selective plates. (B) Following germination of dissected diploids containing heterozygous deletion mutations of *EXO1, NMD2, RAD24* and *CDC13*, non-viable mutants were analyzed using a 20x objective on a Microtec microscope. An image was taken of each microcolony using a Jenoptik camera and all images displayed are reproduced at the same scale for direct comparison.

Figure S3

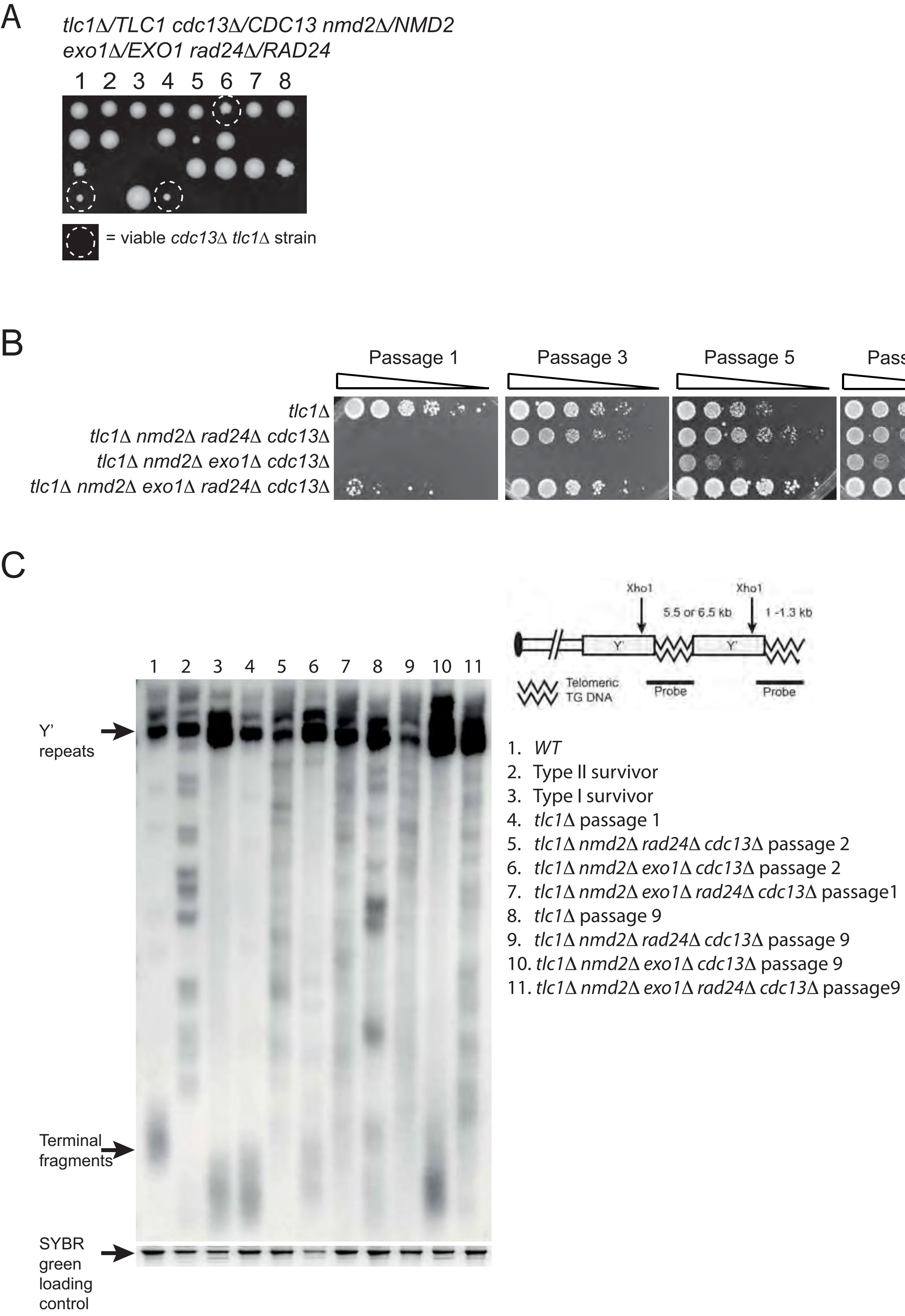
A
tlc1Δ/TLC1 cdc13Δ/CDC13 nmd2Δ/NMD2
exo1Δ/EXO1 rad24Δ/RAD24
1 2 3 4 5 6 7 8
= viable cdc13Δ tlc1Δ strain
B
Passage 1
Passage 3
Passage 5
Passage 7
tlc1Δ
tlc1Δ nmd2Δ rad24Δ cdc13Δ
tlc1Δ nmd2Δ exo1Δ cdc13Δ
tlc1Δ nmd2Δ exo1Δ rad24Δ cdc13Δ
C
Xho1
Xho1
5.5 or 6.5 kb
1 -1.3 kb
Y'
Y'
Telomeric TG DNA
Probe
Probe
1 2 3 4 5 6 7 8 9 10 11
Y' repeats
Terminal fragments
SYBR green loading control
1. WT
2. Type II survivor
3. Type I survivor
4. tlc1Δ passage 1
5. tlc1Δ nmd2Δ rad24Δ cdc13Δ passage 2
6. tlc1Δ nmd2Δ exo1Δ cdc13Δ passage 2
7. tlc1Δ nmd2Δ exo1Δ rad24Δ cdc13Δ passage1
8. tlc1Δ passage 9
9. tlc1Δ nmd2Δ rad24Δ cdc13Δ passage 9
10. tlc1Δ nmd2Δ exo1Δ cdc13Δ passage 9
11. tlc1Δ nmd2Δ exo1Δ rad24Δ cdc13Δ passage9

Figure S3. Viable *cdc13*Δ strains survive without telomerase. Related to Figure 3.

(A) Diploids containing heterozygous deletion mutations of *TLC1*, *EXO1, NMD2, RAD24* and *CDC13* were sporulated. Tetrads were dissected and germinated on YEPD plates. Spores were allowed to form colonies for 5 days at 23°C before being photographed. Genotypes were determined by growth on appropriate selective plates. (B) Strains of the genotypes indicated were repeatedly passaged by toothpick every 4 days at 23°C. At the indicated times 2 ml liquid cultures were grown overnight, serially diluted, spotted onto YEPD plates and incubated for 2 days before being photographed. (C) Genomic DNA was isolated from yeast strains indicated and telomere structures were analyzed by Southern blotting using a Y' and TG probe. SYBR Safe was used as a loading control.

Figure S4

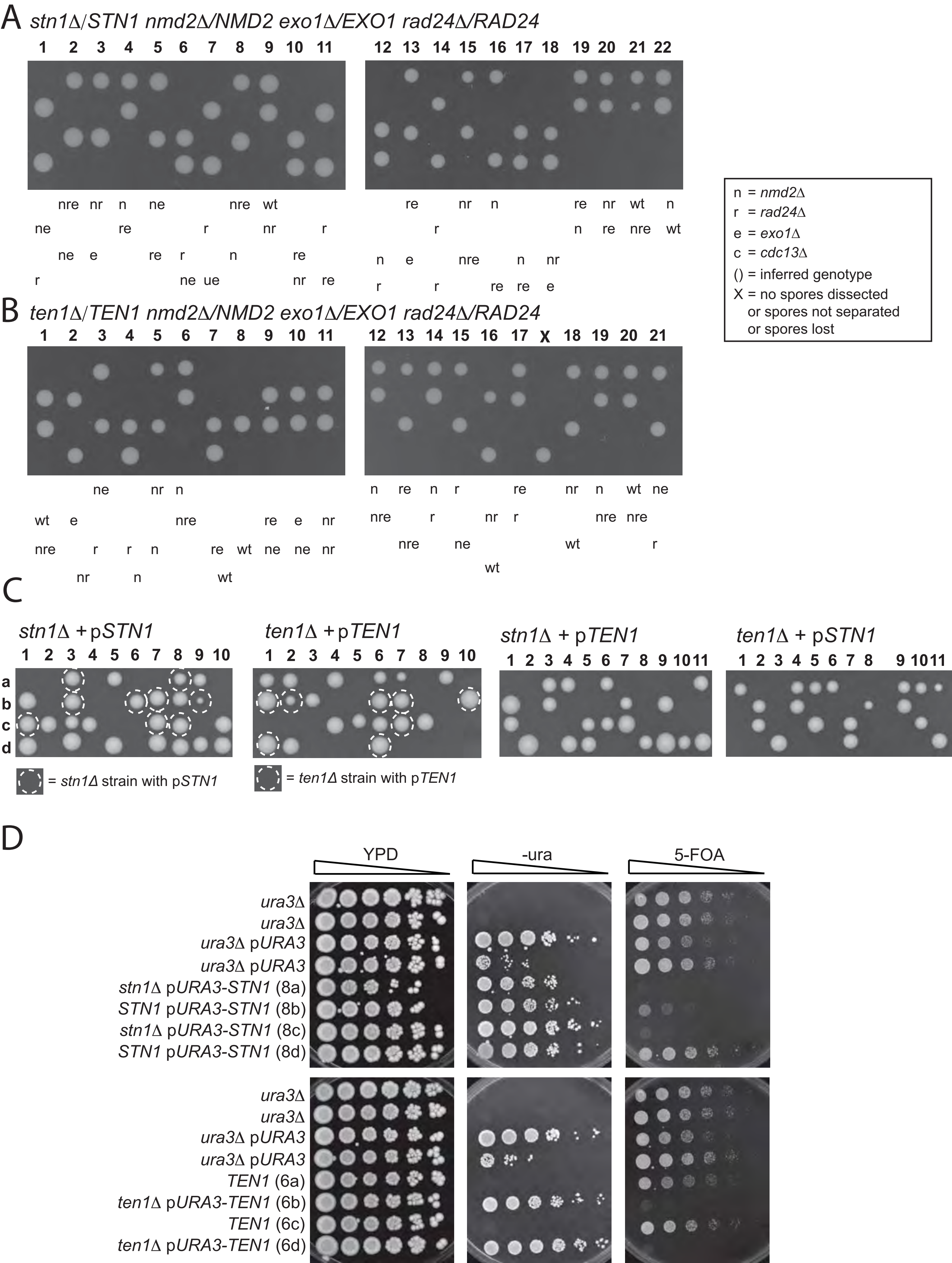

A stn1Δ/STN1 nmd2Δ/NMD2 exo1Δ/EXO1 rad24Δ/RAD24
1 2 3 4 5 6 7 8 9 10 11
12 13 14 15 16 17 18 19 20 21 22
nre nr n ne nre wt re nr n re nr wt n
ne re r nr r r n re nre wt
ne e re r n re n e nre n nr
r ne ue nr re r r re re e
B ten1Δ/TEN1 nmd2Δ/NMD2 exo1Δ/EXO1 rad24Δ/RAD24
1 2 3 4 5 6 7 8 9 10 11
12 13 14 15 16 17 X 18 19 20 21
ne nr n n re n r re nr n wt ne
wt e nre re e nr nre r nr r nre nre
nre r r n re wt ne ne nr nre ne wt r
nr n wt wt
n = nmd2Δ
r = rad24Δ
e = exo1Δ
c = cdc13Δ
() = inferred genotype
X = no spores dissected or spores not separated or spores lost
C
stn1Δ + pSTN1
ten1Δ + pTEN1
stn1Δ + pTEN1
ten1Δ + pSTN1
a
b
c
d
= stn1Δ strain with pSTN1
= ten1Δ strain with pTEN1
D
YPD
-ura
5-FOA
ura3Δ
ura3Δ
ura3Δ pURA3
ura3Δ pURA3
stn1Δ pURA3-STN1 (8a)
STN1 pURA3-STN1 (8b)
stn1Δ pURA3-STN1 (8c)
STN1 pURA3-STN1 (8d)
ura3Δ
ura3Δ
ura3Δ pURA3
ura3Δ pURA3
TEN1 (6a)
ten1Δ pURA3-TEN1 (6b)
TEN1 (6c)
ten1Δ pURA3-TEN1 (6d)

Figure S4. Growth of *stn1*Δ and *ten1*Δ strains. Related to Figure 4.

(A)-(B) Diploids containing heterozygous deletion mutations of *EXO1, NMD2, RAD24* along with deletions of *STN1* or *TEN1* were sporulated. Tetrads were dissected and germinated on YEPD plates. Spores were allowed to form colonies for 5 days at 23°C before being photographed. Genotypes were determined by growth on appropriate selective plates. (C) Diploid yeast strains in (A) and (B) were transformed with linearized plasmid and PCR fragments containing either *STN1* or *TEN1*, to obtain strains expressing *STN1* or *TEN1* on plasmids under their native promoters. Strains were treated as in (A)-(B). (D) Saturated cultures were serially diluted across agar plates containing YPD, synthetic medium without uracil (-ura) or synthetic medium with 5-fluoroorotic acid (5-FOA) and then allowed to form colonies at 30°C for 3 days before being photographed. Strains 8a-d in (D) were *STN1/stn1Δ* strains 8a-d in (C). Strains 6a-d in (B) were *TEN1/ten1Δ* strains 6a-d in (C).

Figure S5

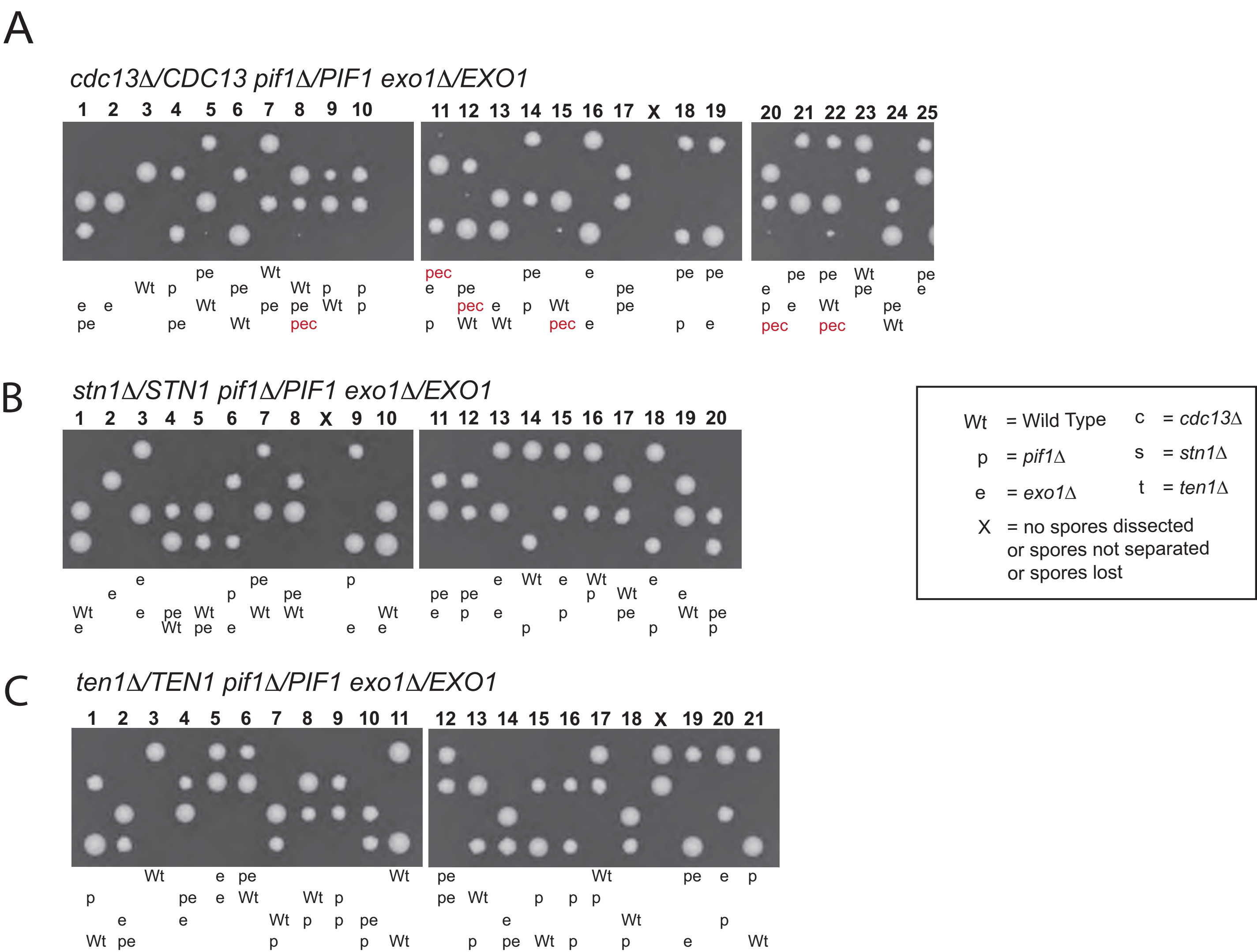
A
cdc13Δ/CDC13 pif1Δ/PIF1 exo1Δ/EXO1
1 2 3 4 5 6 7 8 9 10
11 12 13 14 15 16 17 X 18 19
20 21 22 23 24 25
pe Wt pec pe e pe pe pe pe Wt pe
Wt p pe Wt p p e pe pe e pe e
e e Wt pe pe Wt p pec e p Wt pe p e Wt pe
pe pe Wt pec p Wt Wt pec e p e pec pec Wt
B
stn1Δ/STN1 pif1Δ/PIF1 exo1Δ/EXO1
1 2 3 4 5 6 7 8 X 9 10
11 12 13 14 15 16 17 18 19 20
e pe p e Wt e Wt e
e p pe pe pe p Wt e
Wt e pe Wt Wt Wt Wt e p e p pe Wt pe
e Wt pe e e e p p p
C
ten1Δ/TEN1 pif1Δ/PIF1 exo1Δ/EXO1
1 2 3 4 5 6 7 8 9 10 11
12 13 14 15 16 17 18 X 19 20 21
Wt e pe Wt pe Wt pe e p
p pe e Wt Wt p pe Wt p p p
e e Wt p p pe e Wt p
Wt pe p p Wt p pe Wt p p e Wt
Wt = Wild Type
p = pif1Δ
e = exo1Δ
X = no spores dissected or spores not separated or spores lost
c = cdc13Δ
s = stn1Δ
t = ten1Δ

Figure S5. Bypass of *cdc13*Δ but not *stn1*Δ or *ten1*Δ by *pif1*Δ *exo1*Δ mutations. Related to Figure 5.

(A)-(C) Diploids containing heterozygous deletion mutations of *EXO1* and *PIF1*, along with deletions of *CDC13, STN1* or *TEN1* were sporulated. Tetrads were dissected and germinated on YEPD plates. Spores were allowed to form colonies for 5 days at 23°C before being photographed. Genotypes were determined by growth on appropriate selective plates.

Supplemental Experimental Procedures for

Interactions between CST, DDR and NMD genes show that Stn1 and Ten1 are the key components of the CST complex

Eva-Maria Holstein, Kate R. M. Clark and David Lydall
Correspondence to: David.Lydall@newcastle.ac.uk

This file includes:

1. List of strains used
2. List of primers used
3. List of plasmids used

1. List of yeast strains used. Related figures indicated.

Strain (DLY)	Relevant Genotype	Related Figures
640	*MAT*a	1B, 2G, S1A
1255	*MAT*a *cdc13-1 int rad9::HIS3*	2G, S1A
1272	*MAT*alpha *exo1::LEU2*	5D-E
1273	*MAT*alpha *exo1::LEU2*	5D-E
1296	*MAT*a *exo1::LEU2 cdc13-1 int*	1B, 2G, S1A
1385	*MAT*a *ura3* pRS415*::URA3*	S4D
1468	*MAT*a *cdc13-1 int cdc15-2 bar1::hisG*	2B-E, S1B
1470	*MAT*a *cdc13-1 int rad9::HIS3 cdc15-2 bar1::hisG*	2B-E, S1B
1522	*MAT*a *cdc13-1 int rad24::TRP1*	1B
1628	*MAT*alpha *tlc1::HIS3 pTLC1::URA3*	3C-D
1692	*MAT*a *exo1::LEU2 rad9::HIS3 cdc13-1 int*	2G S1A
1696	*MAT*alpha *cdc13-1 int exo1::LEU2 rad24::TRP1*	1B
2146	*MAT*a *tlc1 ::HIS3*	S3C
2148	*MAT*alpha *tlc1::HIS3 exo1::LEU2*	S3C
3001	*MAT*alpha	5D-E, S4D
4528	*MAT*a *nmd2::HIS3*	6F
4557	*MAT*a *cdc13-1 int*	1B, 2F-G, S1A
4625	*MAT*a *cdc13-1 int nmd2::HIS3*	1B, 2F-G, S1A
4765	*MAT*a *nmd2::HIS3*	1B, 2G, S1A
4766	*MAT*alpha *nmd2::HIS3*	5D-E
4872	*MAT*a *pif1::NATMX*	5D-E
4873	*MAT*alpha *pif1::NATMX*	5D-E
5255	*MAT*alpha *rad9::LEU2 nmd2::HIS3 cdc13-1 int*	2G, S1A
5673	*MAT*a *nmd2::HIS3 rad9::LEU2 cdc13-1 int cdc15-2 bar1::hisG*	2B-E, S1B
5674	*MAT*a *nmd2::HIS3 cdc13-1 int cdc15-2 bar1::hisG*	2B-E, S1B
5750	*MAT*alpha *ura3 pAG36::URA3*	S4D
5759	*MAT*a *STN1-C-MYC::TRP1 nmd2::HIS3*	6B
5761	*MAT*a *STN1-C-MYC::TRP1*	6B
5915	*MAT*a *pif1::NATMX*	5D-E
5916	*MAT*alpha *pif1::NATMX*	5D-E
7273	*MAT*alpha *CDC13-C-MYC::HIS3*	6A
7274	*MAT*a *CDC13-C-MYC::HIS3 nmd2::URA3*	6A
7747	*MAT*a *cdc13-1 int exo1::LEU2 nmd2::URA3*	1B
7749	*MAT*a *cdc13-1 int rad24::TRP1 nmd2::URA3*	1B
7752	*MAT*a *cdc13-1 int rad24::TRP1 exo1::LEU2 nmd2::URA3*	1B
8100	*MAT*a *nmd2::URA3 TEN1-C-MYC::KANMX*	6C
8101	*MAT*a *TEN1-C-MYC::KANMX*	6C
8167	*nmd2::HIS3 exo1::LEU2 cdc13::HPHMX* passage	3C-D

	1	
8168	*nmd2::HIS3 exo1::LEU2 cdc13::HPHMX* passage 1	3D
8169	*nmd2::HIS3 exo1::LEU2 rad24::TRP1 cdc13::HPHMX* passage 1	3C-D
8170	*nmd2::HIS3 exo1::LEU2 rad24::TRP1 cdc13::HPHMX* passage 1	3D
8171	*nmd2::HIS3 rad24::TRP1 cdc13::HPHMX* passage 1	3C-D
8172	*nmd2::HIS3 rad24::TRP1 cdc13::HPHMX* passage 1	3D
8198	*nmd2::HIS3 exo1::LEU2 rad24::TRP1*	6F
8199	*nmd2::HIS3 exo1::LEU2 rad24::TRP1 cdc13::HPHMX* passage 9	3C-D
8200	*nmd2::HIS3 exo1::LEU2 rad24::TRP1 cdc13::HPHMX* passage 9	3D
8201	*nmd2::HIS3 rad24::TRP1 cdc13::HPHMX* passage 9	3C-D
8202	*nmd2::HIS3 rad24::TRP1 cdc13::HPHMX* passage 9	3D
8203	*nmd2::HIS3 exo1::LEU2 cdc13::HPHMX* passage 9	3C-D
8204	*nmd2::HIS3 exo1::LEU2 cdc13::HPHMX* passage 9	3D
8432	*MAT*a *STN1-13MYC::KANMX nmd2::HIS3 exo1::LEU2 rad24::TRP1* passage 8	6D
8434	*MAT*alpha *TEN1-13MYC::KANMX nmd2::HIS3 exo1::LEU2 rad24::TRP1* passage 8	6E
8460	*MAT*a	3C, S3A, 6F
8461	*nmd2::HIS3 exo1::LEU2 rad24::TRP1 cdc13::HPHMX*	6F
8462	*MAT*a *tlc1::NATMX* passage 1	S3B-C
8463	*MAT*a *tlc1::NATMX nmd2::HIS3 rad24::TRP1 cdc13::HPHMX* passage 1	S3B
8464	*MAT*a *tlc1::NATMX nmd2::HIS3 exo1::LEU2 cdc13::HPHMX* passage 1	S3B
8465	*MAT*a *tlc1::NATMX nmd2::HIS3 exo1::LEU2 rad24::TRP1 cdc13::HPHMX* passage 1	S3B-C
8467	*MAT*a *tlc1::NATMX nmd2::HIS3 rad24::TRP1 cdc13::HPHMX* passage 2	S3C
8468	*MAT*a *tlc1::NATMX nmd2::HIS3 exo1::LEU2 cdc13::HPHMX* passage 2	S3C
8472	*MAT*a *tlc1::NATMX* passage 3	S3B
8473	*MAT*a *tlc1::NATMX nmd2::HIS3 rad24::TRP1 cdc13::HPHMX* passage 3	S3B
8474	*MAT*a *tlc1::NATMX nmd2::HIS3 exo1::LEU2 cdc13::HPHMX* passage 3	S3B

8475	*MAT*a *tlc1::NATMX nmd2::HIS3 exo1::LEU2 rad24::TRP1 cdc13::HPHMX* passage 3	S3B
8509	*MAT*a *tlc1::NATMX* passage 5	S3B
8510	*MAT*a *tlc1::NATMX nmd2::HIS3 rad24::TRP1 cdc13::HPHMX* passage 5	S3B
8511	*MAT*a *tlc1::NATMX nmd2::HIS3 exo1::LEU2 cdc13::HPHMX* passage 5	S3B
8512	*MAT*a *tlc1::NATMX nmd2::HIS3 exo1::LEU2 rad24::TRP1 cdc13::HPHMX* passage 5	S3B
8551	*MAT*a *tlc1::NATMX* passage 7	S3B
8552	*MAT*a *tlc1::NATMX nmd2::HIS3 rad24::TRP1 cdc13::hph* passage 7	S3B
8553	*MAT*a *tlc1::NATMX nmd2::HIS3 exo1::LEU2 cdc13::hph* passage 7	S3B
8554	*MAT*a *tlc1::NATMX nmd2::HIS3 exo1::LEU2 rad24::TRP1 cdc13::hph* passage 7	S3B
8600	*MAT*a *tlc1::NATMX* passage 9	S3C
8601	*MAT*a *tlc1::NATMX nmd2::HIS3 rad24::TRP1 cdc13::HPHMX* passage 9	S3C
8602	*MAT*a *tlc1::NATMX nmd2::HIS3 exo1::LEU2 cdc13::hph* passage 9	S3C
8603	*MAT*a *tlc1::NATMX nmd2::HIS3 exo1::LEU2 rad24::TRP1 cdc13::HPHMX* passage 9	S3C
8604	*MAT*alpha *STN1::13MYC::KANMX nmd2::HIS3 exo1::LEU2 rad24::TRP1 cdc13::HPHMX* passage 8	5D
8605	*MAT*a *TEN1::13Myc::KANMX nmd2::HIS3 exo1::LEU2 rad24::TRP1 cdc13::HPHMX* passage 8	5E
**Strain (DDY)**	**Diploid Genotype**	**RELATED FIGURES**
340	*PIF1/pif1::NATMX EXO1/exo1::LEU2 CDC13/cdc13::HPHMX*	5A, S5A
355	*PIF1/pif1::NATMX EXO1/exo1::LEU2 STN1/stn1::HPHMX*	5B, S5B
566	*NMD2/nmd2::HIS3 EXO1/exo1::LEU2 RAD24/rad24::TRP1*	-
568	*NMD2/nmd2::HIS3 EXO1/exo1::LEU2 RAD24/rad24::TRP1 CDC13/cdc13::hph* (DDY 566 transformation)	3A-B, 6D-E,
569	*NMD2/nmd2::HIS3 EXO1/exo1::LEU2 RAD24/rad24::TRP1 STN1/stn1::hph* (DDY 566 transformation)	4A, S4A
571	*NMD2/nmd2::HIS3 EXO1/exo1::LEU2 RAD24/rad24::TRP1 TEN1/ten1::hph* (DDY 566 transformation)	4B
572	*NMD2/nmd2::HIS3 EXO1/exo1::LEU2 RAD24/rad24::TRP1 TEN1/ten1::hph* (DDY 566	4B, S4B

	transformation)	
573	*PIF1/pif1::NATMX EXO1/exo1::LEU2 TEN1/ten1::HPHMX*	5C, S5C
581	*NMD2/nmd2::HIS3 EXO1/exo1::LEU2 RAD24/rad24::TRP1 CDC13/cdc13::HPHMX TLC1/tlc1::NATMX* (DDY568 transformation)	S3A

2. List of oligonucleotides used. Related figures indicated.

Oligo Number	Primer Sequence	Target	Comments (Related Figures)
M417	ATGGGCGCAGGGCCTGGAT TC	Tag for QAOS	Tag/QAOS (Figure 2)
M418	ATGCTCGCAGAGCCCCTGG ATCT	Tag for QAOS	Tag/QAOS (Figure 2)
M1003	AGAGATGCGCCGTTATTGA CGATGCGAGTTTTCTAACAA Agaattcgagctcgtttaaac	*STN1*	For C-terminal Myc-tagging of *STN1* (Figure 6)
M1172	cagaccgaactcggtgattt	*BUD6*	Forward primer qRT-PCR (Figure 5)
M1173	ttttagcgggctgagaccta	*BUD6*	Reverse primer qRT-PCR (Figure 5)
M1509	CAAGAAATGGGAACCTCCA TTAAGATTAGCAA	*RET2*	Reverse primer/QAOS (Figure 2)
M1510	AGCGCACCTGCATCGTTGG CAGCAA	*RET2*	Probe/QAOS (Figure 2)
M1511	ATGGGCGCAGGGCCTGGAT TCATATGGAGCTT	*RET2*	Tagging primer/QAOS (Figure 2)
M1514	TGACTTTCCAAGATGCCTTG AACACTAGTGTC	*DUG1*	Reverse primer/QAOS (Figure 2)
M1515	AATGAGCACCATCATCGCC TCTAC	*DUG1*	Probe/QAOS (Figure 2)
M1516	ATGCTCGCAGAGCCCCTGG ATCTTCATACCACCA	*DUG1*	Tagging primer/QAOS (Figure 2)
M1734	TCGAGCAACTGCAAGAAGA A	*STN1*	Forward primer qRT-PCR (Figure 5)
M1735	CGAAATGACAAGGAATGCA C	*STN1*	Reverse primer qRT-PCR (Figure 5)
M1794	ATACACCAAAGTCCGCCAA T	*TEN1*	Forward primer qRT-PCR (Figure 5)

M1795	CACCAAGTGGTGATTTGAC A	*TEN1*	Reverse primer qRT-PCR (Figure 5)
M1803	CCAACGTCAACGCACC	*TLC1*	To confirm integration of *NATMX* cassette (Figure S3)
M1807	CGTGAGTCTGTGGAATCC	*TLC1*	To confirm integration of *NATMX* cassette (Figure S3)
M1828	CTCTTTGGATACGAATGACC GTGGAAACTATCGCCTAAA ACACATACGATTTAGGTGA CAC	*CDC13*	To integrate hph cassette at *CDC13* locus: M1828, M1829 (Figure 3)
M1829	GCAATTTGGCACCGCCGCG TTGGGCTGCGCGGATCATGT CAATACGACTCACTATAGG GAG	*CDC13*	To integrate hph cassette at *CDC13* locus: M1828, M1829 (Figure 3)
M1897	TCTCCGCCATAGAATCATCA	*CDC13*	To confirm integration: M1897, M1898 (Figure 3)
M1898	GCGTTAAACGTAAATACCT CTTTC	*CDC13*	To confirm integration: M1897, M1898 (Figure 3)
M1943	GCGAAAAAAGAGCAATAAG CTGGCTTTTAATAATGGGTT GGAGCCCACATACGATTTA GGTGACAC	*STN1*	To integrate hph cassette at *STN1* locus: M1943, M1944 (Figure 3)
M1944	CCTAGAGAGATGCGCCGTT ATTGACGATGCGAGTTTTCT AACAAAAATACGACTCACT ATAGGGAG	*STN1*	To integrate hph cassette at *STN1* locus: M1943, M1944 (Figure 3)

M2188	[CY5] CCCACCACACACACCCACA CCC	*TG ssDNA*	Probe/ Fluorescent in-gel assay (Figure 2)
M1367	AATAACGAATTGAGCTATG ACACCAA	*PAC2*	ChIP (Figure 5)
M1368	AGCTTACTCATATCGATTTC ATACGACTT	*PAC2*	ChIP (Figure 5)
M2013	CAATTAAAAGACCTTCTTTG TAGCTTTTAGTGTGATTTTT CTGGTTTGAGCACATACGAT TTAGGTGACAC	*TLC1*	To integrate *NATMX* cassette at *TLC1* locus (Figure S3)
M2014	TGTATATTGTATATTCTAAA AAGAAGAAGCCATTTGGTG GGCTTTATTA AATACGACTCACTATAGGG AG	*TLC1*	To integrate *NATMX* cassette at *TLC1* locus (Figure S3)
M2245	CGTATGCTAAAGTATATATT ACTTCACTCCATT	chrVI-R	ChIP (Figure 4)
M2246	TCCGAACTCAGTTACTATTG ATGGAA	chrVI-R	ChIP (Figure 4)
M2355	ATCACCACTTGGTGAGAAG TTTCTGAACATCTCTAACTC CCGGATCCCCGGGTTAATTA A	*TEN1*	For C-terminal Myc-tagging of *TEN1* (Figure 6)
M2356	AAAGGTATTATATAATCTCA GTATATGCCAAACGTCTGA CGAATTCGAGCTCGTTTAAA C	*TEN1*	For C-terminal Myc-tagging of *STN1* (Figure 6)
M2667	AGCTCTATTTGCTCTTTTTA GAGAACTACGACAGTGTTA TCACATACGATTTAGGTGAC AC	*TEN1*	To integrate *HPHMX* cassette at *TEN1* locus: M2667, M2668 (Figure 3)
M2668	TATCAAAGGTATTATATAAT CTCAGTATATGCCAAACGTC TGACAATACGACTCACTAT AGGGAG	*TEN1*	To integrate *HPHMX* cassette at *TEN1* locus: M2667, M2668 (Figure 3)
M2669	CAGTTGTCGTTATTTTTCTT TT	*TEN1*	To confirm *HPHMX* integration: M2669, M2670 (Figure 3)

M2670	CTTTCCTTTCTTTTCTCATACTTATT	*TEN1*	To confirm *HPHMX* integration: M2669, M2670 (Figure 3)
M2671	CTGCTTGTTGATATTCCATTT	*STN1*	To confirm *HPHMX* integration: M2671, M2672 (Figure 3)
M2672	AATTTTTCAGGCCTAGAGAGAT	*STN1*	To confirm *HPHMX* integration: M2671, M2672 (Figure 3)
M2735	ACCGCGGTGGCGGCCGCATAGGCCACTAGTGGATCTGATATCATCGATGCGATGCTCCGCAGGAATTCT	*STN1*	Amplify and clone into pDL1466: M2735, M2736 (Figure S3)
M2736	CAATTTATAAAGACTGGAGGCAATCACAAACCTCCTGCTCGACGGATAGCCTCTGTCTCTTCTTTCA	*STN1*	Amplify and clone into pDL1466: M2735, M2736 (Figure S3)
M2737	ACCGCGGTGGCGGCCGCATAGGCCACTAGTGGATCTGATATCATCGATGTACCCAAACATATAAATGCC	*TEN1*	Amplify and clone into pDL1466: M2737, M2739 (Figure S3)
M2739	CAATTTATAAAGACTGGAGGCAATCACAAACCTCCTGCTCGACGGATATCTAACCCAGAATTATTGA	*TEN1*	Amplify and clone into pDL1466: M2737, M2739 (Figure S3)
M2752	TGGAATATTATTACATTTGGAATACGCAGCAGCATATTCACGGATCCCCGGGTTAATTAA	*STN1*	For C-terminal Myc-tagging of *STN1*

3. List of plasmids used. Related figures indicated.

Plasmid Number	Details	Source/Alias (Related Figures)
pDL508	kanMX cassette for C-terminal myc-tagging	pFA6a-13Myc-kanMX6. Longtine et al, Yeast 1998 14:953-961. (Figure 6)
pDL987	Cut with *Xho*I and *BamH*I and use 1000bp fragment to detect Y' and telomeric repeat sequences.	pHT128 (pYTEL). Tsubouchi and Ogawa. MBOC. 2000 11:2221 2233 (Figure 3D, Figure S3C)
pDL1389	Plasmid containing hphMX4	pAG32 (Figures 3-4, S3-4)
pDL1221	Plasmid containing natMX4	pAG25 (Figure S3)
pDL1466	Plasmid containing *URA3* and *CAN1*. Cut with *Sph*I and *Asc*I for PCR-mediated gap repair.	Created by Marion Dubarry (Newcastle University); derivative of pDL1279 (Figure S3)
